# Reprogramming of PD-1^+^ M2-like tumor-associated macrophages with anti–PD-L1 and lenalidomide in cutaneous T cell lymphoma

**DOI:** 10.1172/jci.insight.163518

**Published:** 2023-07-10

**Authors:** Zhen Han, Xiwei Wu, Hanjun Qin, Yate-Ching Yuan, Daniel Schmolze, Chingyu Su, Jasmine Zain, Lilach Moyal, Emmilia Hodak, James F. Sanchez, Peter P. Lee, Mingye Feng, Steven T. Rosen, Christiane Querfeld

**Affiliations:** 1 Division of Dermatology,; 2 Beckman Research Institute,; 3 Department of Computational and Quantitative Medicine,; 4 Integrative Genomics Core,; 5 Center for informatics,; 6 Department of Pathology, and; 7 Department of Hematology and Hematopoietic Cell Transplantation, City of Hope, Duarte, California, USA.; 8 Department of Dermatology, Rabin Medical Center, Felsenstein Medical Research Center, Tel Aviv University, Tel Aviv, Israel.; 9 Beilinson Hospital, Sackler Faculty of Medicine, Tel Aviv University, Tel Aviv, Israel.; 10 Department of Immuno-Oncology, City of Hope, Duarte, California, USA.

**Keywords:** Dermatology, Cancer immunotherapy, Lymphomas, Macrophages

## Abstract

Cutaneous T cell lymphoma (CTCL) is a disfiguring and incurable disease characterized by skin-homing malignant T cells surrounded by immune cells that promote CTCL growth through an immunosuppressive tumor microenvironment (TME). Preliminary data from our phase I clinical trial of anti–programmed cell death ligand 1 (anti–PD-L1) combined with lenalidomide in patients with relapsed/refractory CTCL demonstrated promising clinical efficacy. In the current study, we analyzed the CTCL TME, which revealed a predominant PD-1^+^ M2-like tumor-associated macrophage (TAM) subtype with upregulated NF-κB and JAK/STAT signaling pathways and an aberrant cytokine and chemokine profile. Our in vitro studies investigated the effects of anti–PD-L1 and lenalidomide on PD-1^+^ M2-like TAMs. The combinatorial treatment synergistically induced functional transformation of PD-1^+^ M2-like TAMs toward a proinflammatory M1-like phenotype that gained phagocytic activity upon NF-κB and JAK/STAT inhibition, altered their migration through chemokine receptor alterations, and stimulated effector T cell proliferation. Lenalidomide was more effective than anti–PD-L1 in downregulation of the immunosuppressive IL-10, leading to decreased expression of both PD-1 and PD-L1. Overall, PD-1^+^ M2-like TAMs play an immunosuppressive role in CTCL. Anti–PD-L1 combined with lenalidomide provides a therapeutic strategy to enhance antitumor immunity by targeting PD-1^+^ M2-like TAMs in the CTCL TME.

## Graphical abstract

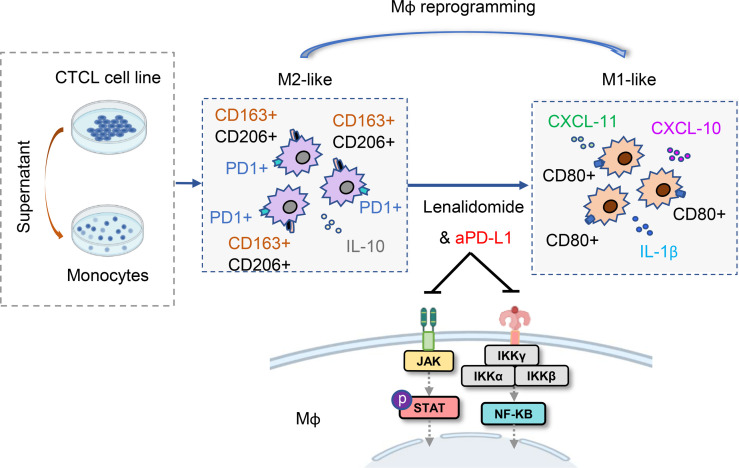

## Introduction

Mycosis fungoides (MF) and the leukemic variant Sézary syndrome (SS) comprise the most common types of cutaneous T cell lymphomas (CTCLs) that develop from skin-homing clonally expanded CD4^+^ T cells in a background of chronic inflammation (1). Although the pathogenesis of CTCL is largely unknown, a variety of growth factors, cytokines, and chemokines are reported to be involved in the development of CTCL (2–6). Macrophages are important regulators in the tumor microenvironment (TME) by presenting antigens to T cells and having key roles in tumor immune surveillance and tolerance, thereby showing plasticity in producing distinct cytokines and chemokines that can influence tumor cell migration and proliferation in the TME. Tumor-associated macrophages (TAMs) exhibit a CD163^+^ M2 macrophage phenotype and are abundant in skin lesions of MF (7–9). The presence of TAMs with a CD163^+^ M2 phenotype has been associated with disease progression in patients with CTCL (8–10). In addition, depletion of CD163^+^ M2-like TAMs in CTCL xenograft mouse models decreased tumor growth, which further supports their critical role in CTCL pathogenesis (9).

The programmed cell death 1/programmed cell death ligand 1 (PD-1/PD-L1) pathway is utilized to suppress antitumor immune responses and to evade immune surveillance (11–13). We and others have shown that CTCL cells escape immune surveillance via immune checkpoint signaling such as the PD-1/PD-L1 axis (14, 15). In addition to T cells (16–18), PD-1 expression on other cell types including TAMs (19–21) and DCs (22–24) has been shown, which may impair immunity. Gordon et al. demonstrated that PD-1 expression in TAMs increased over time in mouse models of cancer and with increasing disease stage in human cancers and that the PD-1/PD-L1 axis blockade increased the antitumor efficacy of TAMs (19). PD-1 expression in TAMs can be induced by multiple cytokines via STAT and NF-κB signaling pathways (21, 25). These findings support the notion that PD-1/PD-L1 blockade may enhance antitumor immunity by inhibiting immunosuppressive TAMs and cytokines in addition to reversing T cell exhaustion/dysfunction toward an effective immune response (26). Although PD-1/PD-L1 blockade has been used for CTCL and the anti–PD-L1 inhibitor durvalumab is currently evaluated in a phase I/II clinical trial in combination with lenalidomide in patients with refractory/relapsed CTCL (27) (ClinicalTrials.gov NCT03011814), little is known about their biological effects on TAMs in the CTCL TME (28).

Here, we have investigated the role of PD-1 expression on TAMs, particularly on M2-like TAMs and their impact on the CTCL TME. In addition, we studied the therapeutic effects of the PD-L1 inhibitor durvalumab alone or when combined with lenalidomide on the CTCL TME.

## Results

### Accumulation of M2-like TAMs in lesional skin of CTCL shows PD-1 expression.

**Figure 1:**
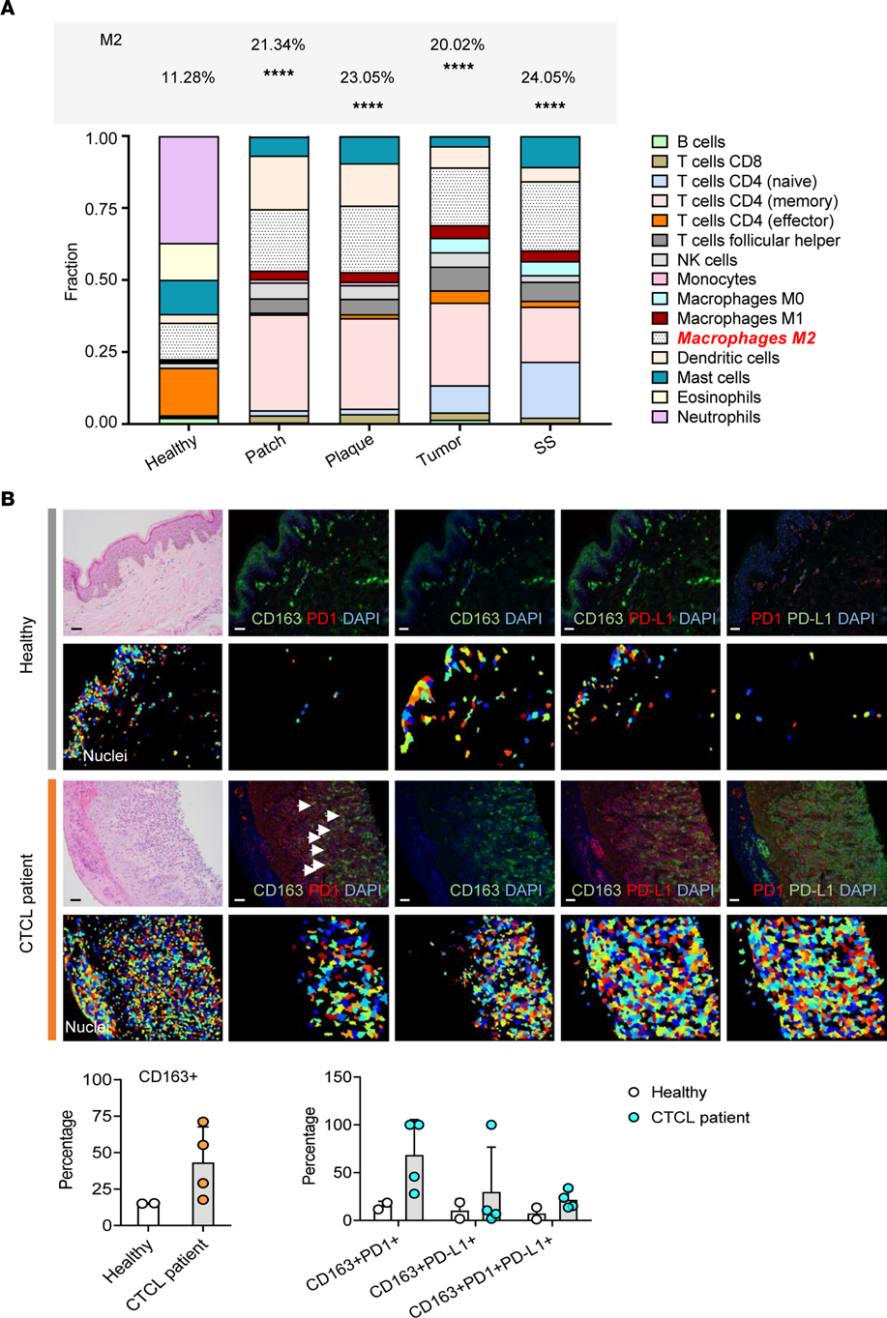
M2-like TAMs are abundant within the TME of CTCL. (**A**) CIBERSORT analysis of the immune cell profile in patients with CTCL (patch, *n* =7; plaque, *n* = 16; tumor, *n* = 18; and SS, *n* = 4) and healthy control (*n* = 3) are shown as stacked bar graph. Data are representative of 3 independent experiments. Significant difference was determined by 1-way ANOVA and *P* ≤ 0.05 was considered significant. ****P < 0.0001. (**B**) Representative H&E and immunofluorescence (IF) staining shows the presence of PD-1^+^ M2-like TAMs in CTCL and in healthy control (top row shows the original IF image; the bottom row shows the analyzed image using CellProfiler). Scale bar: 100 μm. Statistical analysis of IF staining shows the fraction of PD-1^+^CD163^+^ cells, PD-L1^+^CD163^+^ cells, and PD-1^+^PD-L1^+^ cells in CTCL (*n* = 4) and compared with healthy control (*n* = 2). Data are representative of 3 independent experiments with mean ± SD. Significant difference was determined by Pearson’s χ^2^ test, and *P* ≤ 0.05 was considered significant.

We first assessed the Hematology B37 database (QIAGEN Digital Insights) to determine whether gene signatures for M2-like TAMs, defined by CD163 expression, are increased in hematologic malignancies. The fragments per kilobase of transcript per million mapped reads values of CD163 expression in CTCL skin samples and other hematologic malignancies are shown in Supplemental Figure 1A (supplemental material available online with this article; https://doi.org/10.1172/jci.insight.163518DS1). CTCL has the highest gene signature for *CD163* compared with other hematologic malignancies and to healthy controls (*P* < 0.0001). In addition, we used CIBERSORT to quantify immune cell fractions from gene expression profiles of 45 lesional CTCL specimens and 3 healthy skin controls for immune cell gene clusters including macrophage subsets, T cell subsets, monocytes, DCs, mast cells, eosinophils, neutrophils, B cells, and NK cells. The different immune cell gene contents in the TME stratified by lesion type are depicted in Figure 1A. Notably, M2-like macrophage genes were upregulated in patch, plaque, tumor, and erythrodermic/SS lesions compared with healthy control (*P* < 0.0001), suggesting a significant shift toward M2-like TAMs. Our RNA-Seq data analysis revealed upregulated expression for *CD163* (*P* < 0.0001), *PDCD1* (*P* < 0.001), and *CD274* (*P* < 0.0001) in lesional CTCL skin samples compared with that in normal controls (Supplemental Figure 1B). Since the panmacrophage marker CD68 and M2 macrophage marker CD163 are often coexpressed, we correlated the gene expression profile of CD68 with CD163. We found that the *CD163* gene positively correlated with *CD68* (*r* = 0.61, *P* < 0.0001, *n* = 45) and the M2 macrophage marker *MRC1* (CD206) (*r* = 0.7, *P* < 0.0001, *n* = 45), but not with the M1 markers *CD80*, *CD86*, and *SOCS1* (*r* = 0.116, *P* > 0.05; *r* = 0.39, *P* > 0.05; *r* = 0.32, *P* > 0.05; *n* = 45) (Supplemental Figure 1, C and D). Furthermore, we performed IHC staining for CD163, PD-1, and PD-L1 on skin sections from lesional CTCL specimens and healthy controls. In normal skin, very few CD163^+^ and PD-1^+^ cells were detected in the dermis. However, high numbers of CD163^+^ and PD-1^+^ cells were populated in the dermis of the CTCL lesional skin. We also analyzed for PD-L1^+^/CD163^+^ and PD-1^+^/PD-L1^+^ coexpressing cells compared with normal skin. The percentage of PD-1^+^/CD163^+^ coexpressing cells in lesional CTCL sections was higher compared with PD-L1^+^/CD163^+^ and PD-1^+^/PD-L1^+^ coexpression (Figure 1B). Taken together, the data support a predominant PD-1^+^ M2-like TAM phenotype in the CTCL microenvironment.

### Aberrant cytokine profile in CTCL correlates with M2-like TAM polarization.

**Figure 2:**
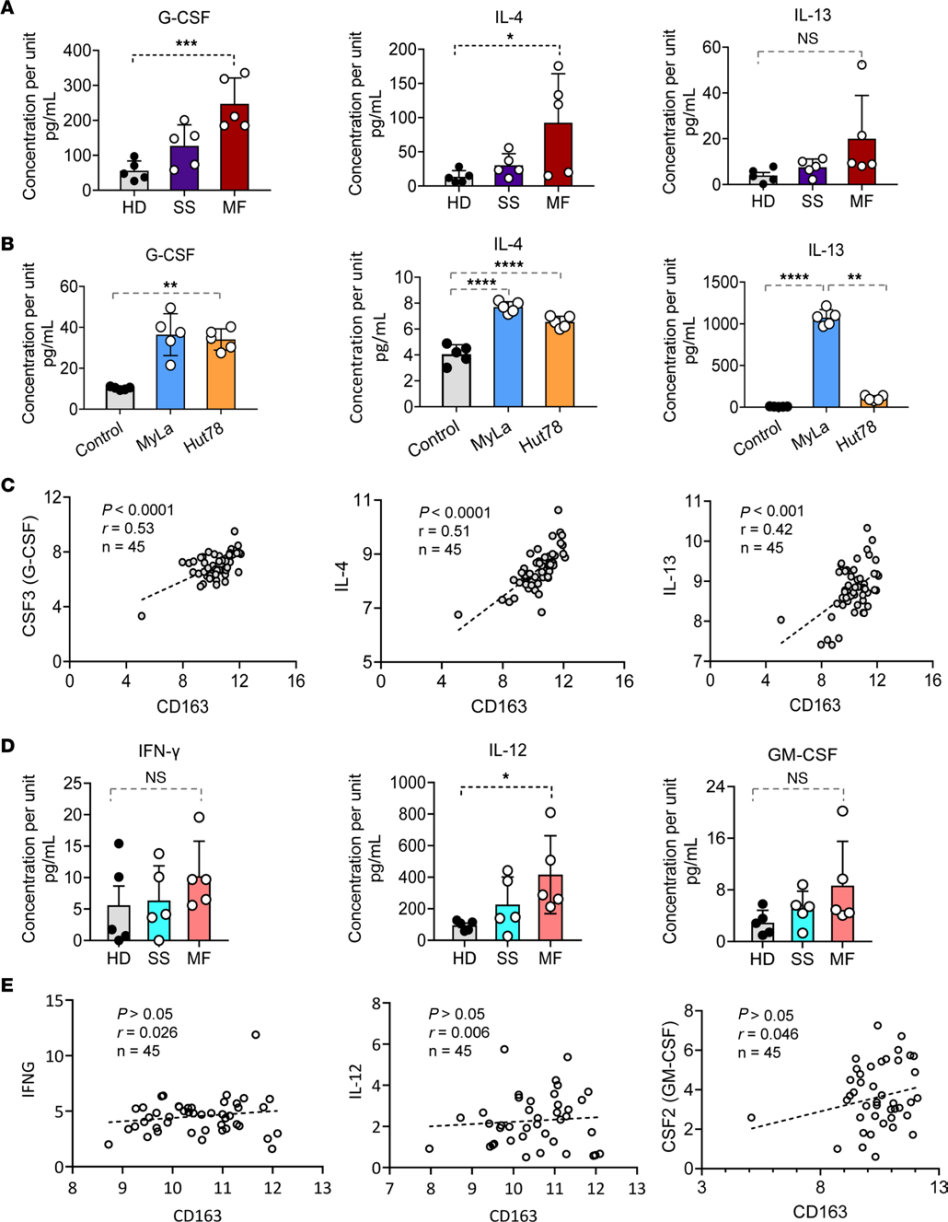
Th2 cytokine profile in patients with CTCL correlates with M2-like TAMs. (**A**) Luminex cytokine analysis for the Th2-cytokines G-CSF, IL-4, and IL-13 in plasma samples of patients with MF and SS and in HDs. Data are representative of 3 independent experiments with mean ± SD. Significant difference was determined by 1-way ANOVA. *P < 0.05, ***P < 0.001, *n* = 5. (**B**) Luminex cytokine analysis for G-CSF, IL-4, and IL-13 in MyLa and Hut78 culture medium and control medium (*n* = 5). Data are representative of 3 independent experiments with mean ± SD. Significant difference was determined by 1-way ANOVA and *P* ≤ 0.05 was considered significant. **P < 0.01, ****P < 0.0001. (**C**) RNA-Seq gene expression values of *CSF3*, *IL-4*, and *IL-13* in lesional CTCL skin samples positively correlated with *CD163* (*n* = 45). Data are representative of 3 independent experiments. The Spearman’s correlation coefficient was determined (*r* = 0.53, ****P < 0.0001; *r* = 0.51, ****P < 0.0001; *r* = 0.42, ***P < 0.001; *n* = 45). (**D**) Luminex cytokine analysis for IFN-γ, IL-12, and GM-CSF in plasma samples of patients with MF and SS and in HD. Data are representative of 3 independent experiments with mean ± SD. *n* = 5. Significant difference was determined by 1-way ANOVA and *P* ≤ 0.05 was considered significant. *P < 0.05. (**E**) Correlation analysis of RNA-Seq gene expression values of *IFNG*, *IL-12*, and *CSF2* (GM-CSF) in lesional CTCL skin samples with *CD163* (*n* = 45). Data are representative of 3 independent experiments. The Spearman’s correlation coefficient was determined (*r* = 0.026, *P* > 0.05; *r* = 0.006, *P* > 0.05; *r* = 0.046, *P* > 0.05; *n* = 45).

We measured plasma cytokines from patients with CTCL and healthy controls. Plasma G-CSF (*P* < 0.01), IL-4 (*P* < 0.05), and IL-13 (NS) levels, known to be associated with M2-like TAM polarization (8), were significantly higher in patients with MF compared with levels in healthy controls (Figure 2A). The same cytokine profile (G-CSF, IL-4, and IL-13) was assessed in CTCL cell line (MyLa, Hut78) supernatants, which also demonstrated higher levels of G-CSF (NS, *P* < 0.01), IL-4 (*P* < 0.0001, *P* < 0.0001), and IL-13 (*P* < 0.0001, *P* < 0.01) compared with that in culture medium alone (control) (Figure 2B). The results indicated that the cytokine profile in supernatants of CTCL cell lines was similar to plasma samples of CTCL patients. RNA-Seq analysis revealed that the gene level of *CD163* positively correlated with *CSF3* (G-CSF, *r* = 0.53, *P* < 0.0001, *n* = 45), *IL-4* (*r* = 0.51, *P* < 0.0001, *n* = 45), and *IL-13* (*r* = 0.42, *P* < 0.001, *n* = 45) in CTCL (Figure 2C), suggesting M2-like TAMs are induced and promoted by these cytokines. In addition, we analyzed plasma for IFN-γ, IL-12, and GM-CSF levels, known to be associated with M1-like TAM polarization. Although these cytokines were elevated in MF samples compared with healthy controls (Figure 2D), *CD163* gene expression did not positively correlate with *IFNG* (*r* = 0.026, *P* > 0.05, *n* = 45), *IL-12* (*r* = 0.006, *P* > 0.05, *n* = 45), and *CSF2* gene expression (GM-CSF, *r* = 0.046, *P* > 0.05, *n* = 45) (Figure 2E), suggesting that M2-like TAMs are not induced or promoted by IFN-γ, IL-12, and GM-CSF. To identify the cellular source of those cytokines, we performed CIBERSORT analysis from the skin biopsy RNA-Seq data. The data demonstrated that the G-CSF, IL-4, and IL-13 gene expression profile was induced in M2-like TAMs but not in M1-like phenotypes in the CTCL TME. While IFNG, IL-12, and GM-CSF expression was seen in various immune cell types, IL-12 was expressed in M1-like TAMs but not in M2 (Supplemental Figure 2A). The standard curve ranges for the related cytokines are shown in Supplemental Figure 2, B–D.

### Chemokine expression profiles in CTCL correlate with accumulation of M2-like TAMs in the CTCL microenvironment.

**Figure 3:**
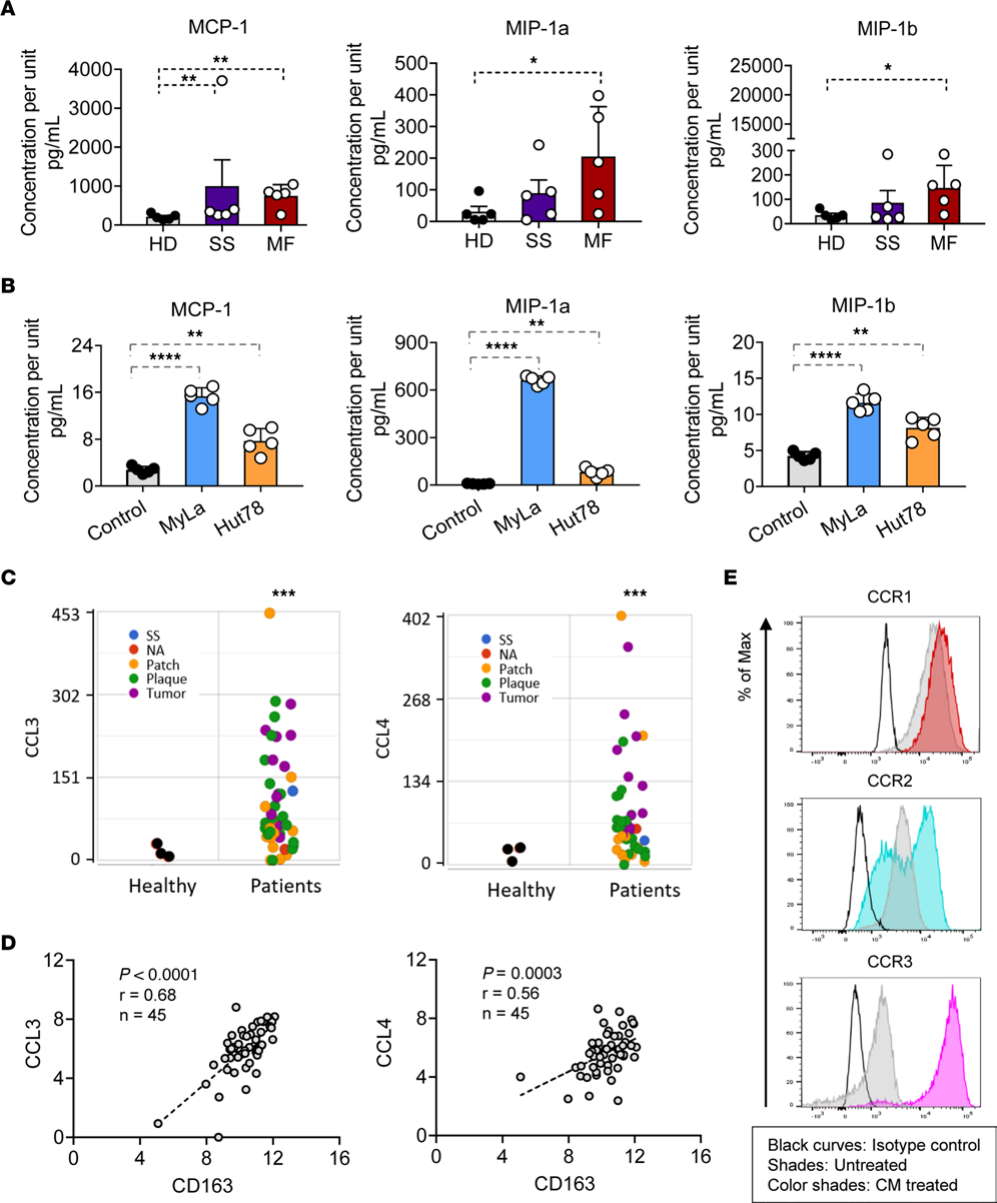
Increased levels of chemokines CCL2, CCL3, and CCL4 in patients with CTCL correlate with M2-like TAMs. (**A**) The concentrations of MCP-1 (CCL2), MIP-1α (CCL3), and MIP-1β (CCL4) in plasma samples (*n* = 5) compared with HD (*n* = 5). (**B**) The concentrations of MCP-1, MIP-1α, and MIP-1β in MyLa (*n* = 5) and Hut78 (*n* = 5) supernatant compared with blank culture medium. Data are representative of 3 independent experiments with mean ± SD for **A** and **B**. Significant difference was determined by 1-way ANOVA and *P* ≤ 0.05 was considered significant. *P < 0.05; **P < 0.01; ****P < 0.0001. (**C**) RNA-Seq gene-level analysis plots indicate the expression levels of *CCL3* and *CCL4* genes in CTCL (*n* = 45) and normal controls (*n* = 3). Data are representative of 3 independent experiments. The green dots indicate plaque, yellow dots show patch, purple dots show tumor, blue dots represent SS, and black dots represent normal. ***P < 0.001, by 2-tailed Student’s *t* test. (**D**) Lesional CTCL skin RNA-Seq gene expression levels of *CCL3* and *CCL4* positively correlated with *CD163* (*n* = 45). Data are representative of 3 independent experiments. The Spearman’s correlation coefficient was determined (*r* = 0.68, *P* < 0.0001; *r* = 0.56, *P* < 0.0003). (**E**) The expression levels of CCR1, CCR2, and CCR5 were detected on macrophages induced by CTCL cell line supernatant using flow cytometry. The histograms were representative of 3 independent experiments. The black curves represent the fluorescence intensity of the isotype control, shades represent the fluorescence intensity of untreated, and color lines represent the fluorescence intensity of CM treated.

Chemokine and chemokine receptor interactions that facilitate the recruitment of monocytes to the skin are well documented. Monocyte chemoattractant protein–1 (MCP-1/CCL2), macrophage inflammatory protein–1α (MIP-1α/CCL3), and MIP-1β (MIP-1β/CCL4) are the key chemokines that regulate macrophage trafficking. The expression of plasma chemokines MCP-1 (*P* < 0.01), MIP-1α (*P* < 0.05), and MIP-1β (*P* < 0.05) was increased in CTCL samples compared with levels in healthy controls (Figure 3A). Similar chemokine profiles were seen in supernatants of CTCL cell lines (MyLa, HuT78) with higher levels noted in CTCL cell line supernatants MCP-1 (*P* < 0.0001, *P* < 0.01), MIP-1α (*P* < 0.0001, *P* < 0.01), and MIP-1β (*P* < 0.0001, *P* < 0.01) compared with that in control medium (Figure 3B). RNA-Seq profiling of lesional CTCL samples (*n* = 45) revealed overexpression of *CCL2* (NS), *CCL3* (*P* < 0.001), and *CCL4* (*P* = 0.001) when compared with healthy controls (Figure 3C and Supplemental Figure 3A). In addition, the results demonstrated a positive correlation for CCL3 and CCL4 with CD163 (Spearman’s correlation coefficient: *r* = 0.68, *P* < 0.0001, *n* = 45; *r* = 0.56, *P* < 0.0003, *n* = 45; Figure 3D), but not for CCL2 (Supplemental Figure 3B), suggesting that CCL3 and CCL4 may facilitate the recruitment and infiltration of M2-like TAMs in CTCL. The chemokine ligands must combine with their corresponding receptor to function; thus, the relative levels of CCR1, CCR2, and CCR5 were investigated on M2-like TAMs when cultured in CTCL-conditioned medium. We found that all 3 chemokine receptors were highly expressed on CTCL-conditioned medium–induced M2-like TAMs compared with TAMs without conditioned medium (Figure 3E and Supplemental Figure 3, C and D). We also analyzed our skin biopsy RNA-Seq data for the chemokine and corresponding receptor CCL2 (CCR1), CCL3 (CCR2), and CCL4 (CCR5) expression profile for each immune cell cluster by CIBERSORT. Notably, all these chemokines and their receptors CCR1, CCR2, and CCR5 were highly expressed on M2-like TAMs (Supplemental Figure 4A). The results suggested the chemokine profile in CTCL may contribute to recruitment of their relevant receptor-positive M2-like TAMs toward an immunosuppressive TME. The standard curve ranges for each corresponding chemokine are shown in Supplemental Figure 4, B–D.

### Activation of NF-κB and JAK/STAT signaling in primary and in M2-like TAMs trained in vitro.

**Figure 4:**
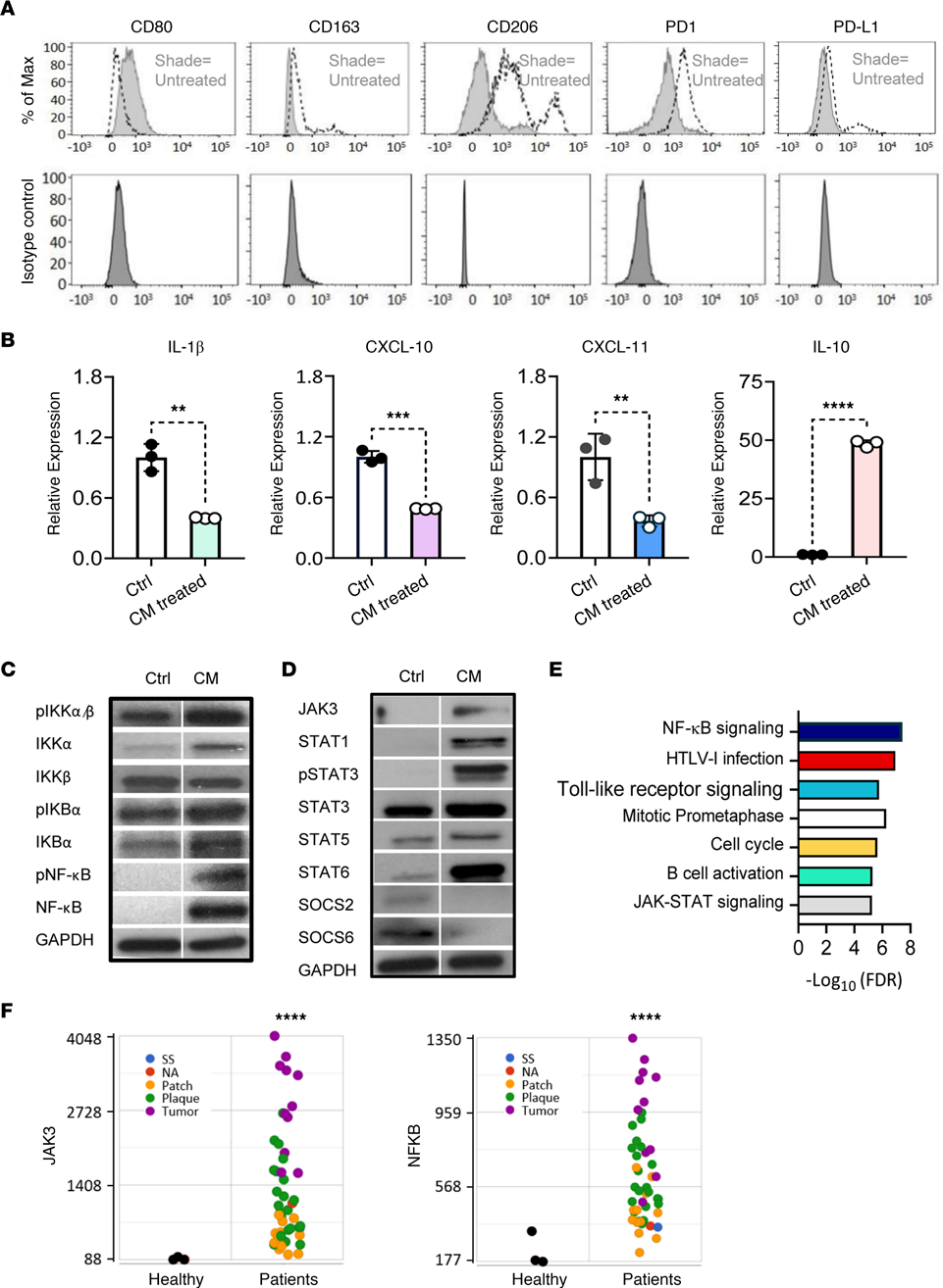
CTCL cell line supernatant induces PD-1^+^ M2-like TAMs in vitro. Healthy CD14^+^ monocytes were cultured for 72 hours in CTCL cell line–conditioned medium. (**A**) The expression of CD80, CD163, CD206, PD-1, and PD-L1 was detected in TAMs induced by CTCL cell line supernatant using flow cytometry. The histograms were representative of 3 independent experiments. The lighter shades at the top represent the fluorescence intensity of the untreated controls, and the darker shades at the bottom represent the fluorescence intensity of the isotype controls. (**B**) IL-1β, CXCL-10, CXCL-11, and IL-10 mRNA levels were assessed in total CD14^+^ cells cultured with or without CTCL cell line supernatant. Data are presented as mean ± SD from 3 biological replicates. **P < 0.01, ***P < 0.001, ****P < 0.0001, by 2-tailed Student’s *t* test. (**C**) Protein expression of p-IKKα/β, IKKα, IKKβ, p–NF-κB, IKBα, p-IKBα, and NF-κB and (**D**) JAK3, STATs, and SOCSs in lysates from TAMs induced by CM compared with expression from monocytes without CM assessed by Western blotting using GAPDH as a loading control (Ctrl, control; CM, CTCL cell line supernatant). One representative image of 3 independent samples per group is shown. (**E**) The hallmark pathway analysis of differentially expressed genes in CTCL (*n* = 45) as compared with healthy controls (*n* = 45). Data are representative of 3 independent experiments. For each pathway shown, the difference between groups had an FDR *q* value less than 0.05. (**F**) RNA-Seq gene-level analysis plots reveal the expression levels of *JAK3* and *NF-κB* genes in CTCL (*n* = 45) and healthy controls (*n* = 3). The green dots indicate plaque, yellow dots indicate patch, purple dots represent tumor, blue dots show SS, and black dots show normal. Data are representative of 3 independent experiments. ****P < 0.0001, by 2-tailed Student’s *t* test.

Human peripheral blood-derived CD14^+^ monocytes from healthy donors (HDs) cultured in CTCL cell line (MyLa or Hut78) supernatants were analyzed for the prototypical M1 marker CD80, the M2 markers CD163 and CD206, and the immune checkpoint proteins PD-1 and PD-L1 and their expression level was quantitated by flow cytometry. TAMs exhibited an M2 phenotype with high levels of PD-1 expression and demonstrated strong induction of CD163 and CD206, but they showed downregulation of CD80 compared with untreated control (Figure 4A and Supplemental Figure 5). The mRNA expression of cytokine/chemokine profiles associated with M1 or M2 activation states was assessed by reverse transcription PCR (RT-PCR). TAMs induced by CTCL cell line supernatants expressed high levels of IL-10 and significantly lower levels of proinflammatory cytokine/chemokines IL-1β (*P* < 0.01), CXCL-10 (*P* < 0.001), and CXCL-11 (*P* < 0.01) compared with untreated control (Figure 4B). We therefore assessed CTCL cell line supernatant-induced M2-like TAMs for activated transduction proteins involved in the NF-κB signaling (Figure 4C) and JAK/STAT signaling cascade (Figure 4D) using Western blot. The quantitated results revealed activated phosphorylated (p-) IKKα, IKKα, p–NF-κB, NF-κB, JAK3, STAT1, p-STAT3, STAT5, and STAT6 and downregulated SOCS2 and SOCS6 (Supplemental Figures 6 and 7). Our results suggest that activated NF-κB and JAK/STAT signaling mediate TAM polarization toward the M2 phenotype. Moreover, RNA-Seq analysis of lesional CTCL skin revealed activated signaling pathways in CTCL compared with HD, including NF-κB and JAK/STAT signaling (Figure 4E), and highlighted upregulation of *JAK3* (*P* < 0.0001) and *NF-κB* (*P* < 0.0001) genes with highest expression in tumor lesions compared with HD (Figure 4F). Additionally, *CD163* positively correlated with *JAK3* (*r* = 0.59, *P* < 0.0001, *n* = 45), *NF-κB* (*r* = 0.47, *P* < 0.0001, *n* = 45), and *STAT6* (*r* = 0.38, *P* < 0.01, *n* = 45) but negatively correlated with *SOCS6* (*r* = 0.32, *P* < 0.05, *n* = 45) (Supplemental Figure 8, A–D), suggesting that JAK/STAT and NF-κB are involved in M2-like TAM programming in the CTCL microenvironment.

### Anti–PD-L1 and lenalidomide lead to functional remodeling of tumor-associated macrophages from M2- to M1-like phenotypes through downregulation of PD-1 and PD-L1 upon NF-κB and JAK/STAT inhibition.

**Figure 5:**
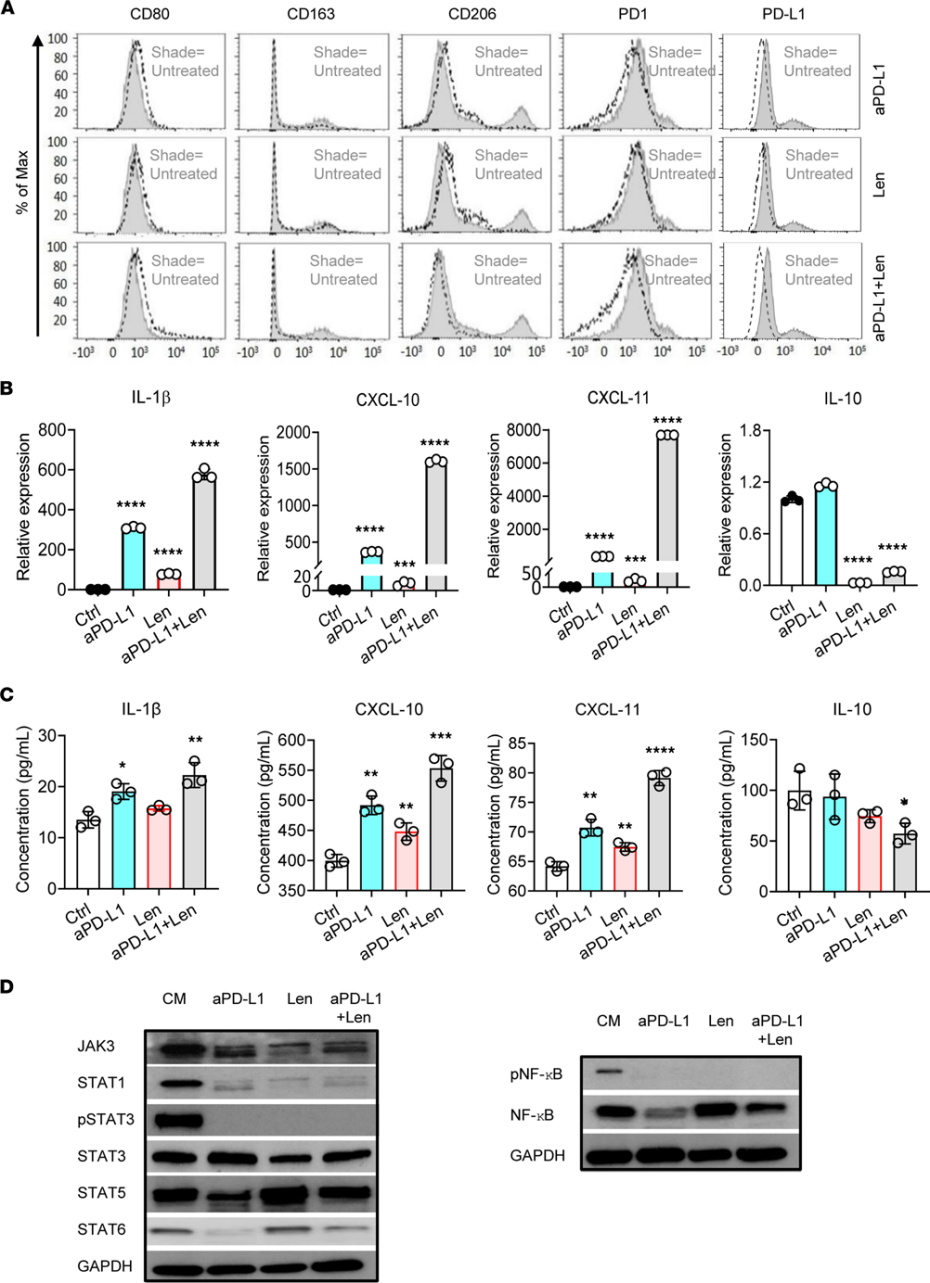
Lenalidomide and anti–PD-L1 Ab reprogram M2-like TAMs in vitro. (**A**) CTCL cell line supernatant-induced TAMs were treated with anti–PD-L1 (10 μg/mL), lenalidomide (10 μM), or their combination for 72 hours. The expression of CD80, CD163, CD206, PD-1, and PD-L1 was detected by flow cytometry. The histograms are representative of 3 independent experiments. The shades represent the fluorescence intensity of the untreated control. (**B**) IL-1β, CXCL-10, CXCL-11, and IL-10 mRNA levels were assessed in total CD14^+^ cells cultured with CTCL cell line supernatant and treated with control, anti–PD-L1, lenalidomide, or combined anti–PD-L1 and lenalidomide. (**C**) IL-1β, CXCL-10, CXCL-11, and IL-10 protein levels in supernatants were assessed by ELISA in anti–PD-L1, lenalidomide, combined anti–PD-L1 and lenalidomide treatment, or untreated control. Data are representative of 3 independent experiments with mean ± SD for **B** and **C**. Significant difference was determined by 1-way ANOVA and *P* ≤ 0.05 was considered significant. *P < 0.05; **P < 0.01; ***P < 0.001; ****P < 0.0001. (**D**) Western blot was utilized to determine the levels of JAK3, STATs, p–NF-κB, NF-κB, and GAPDH in lysates from CTCL cell line supernatant-induced TAMs that were untreated or treated with anti–PD-L1, lenalidomide, or their combination. Results show a representative image from 3 independent samples per group.

To investigate whether anti–PD-L1 and lenalidomide (IC_50_ = 10.12 μM, Supplemental Figure 9) modulate M2-like TAM polarization cultured in conditioned medium with CTCL cell line supernatant, we analyzed and quantitated the expression profiles for the prototypical M1 marker CD80 and the M2 markers CD163 and CD206 by flow cytometry. The data indicated induction of CD80 (Figure 5A) and CD86 (Supplemental Figure 10A) consistent with an M1 phenotype and downregulation of CD163, CD206, PD-1, and PD-L1 after treatment. Synergistic effects were noted when both drugs were combined (Figure 5A and Supplemental Figure 10B). To determine whether anti–PD-L1 (durvalumab), lenalidomide, or the combination influences the cytokine/chemokine gene expression in CTCL cell line supernatant-induced M2-like TAMs, we isolated total RNA after treatment, and expression of the IL-1β, CXCL-10, CXCL-11, and IL-10 genes was analyzed by RT-PCR and their protein levels in supernatants by ELISA. The results show that anti–PD-L1 and lenalidomide synergistically increased IL-1β, CXCL-10, and CXCL-11 expression but significantly decreased IL-10 levels when compared with untreated control, and amplified levels were noted when both drugs were combined (Figure 5, B and C). The changes both at mRNA and protein level demonstrated the functional changes that occur with M1-like polarization.

Western blot analysis and quantitation revealed that JAK3, STAT1, p-STAT3, STAT5, STAT6, p–NF-κB, and NF-κB expression was decreased after anti–PD-L1, lenalidomide, or their combination as compared with untreated control (Figure 5D and Supplemental Figure 11, A and B). Our results indicated that anti–PD-L1 and lenalidomide blunted the JAK/STAT and NF-κB activation cascade, subsequently leading to M1 polarization with upregulation of proinflammatory cytokines/chemokines IL-1β, CXCL-10, and CXCL-11 and downregulation of immunosuppressive PD-1 and PD-L1 expression.

### Anti–PD-L1 and lenalidomide inhibit migration of M2-like TAMs through downregulation of chemokines and promote macrophage-mediated phagocytosis and T cell proliferation to promote antitumor immunity in the CTCL TME.

**Figure 6:**
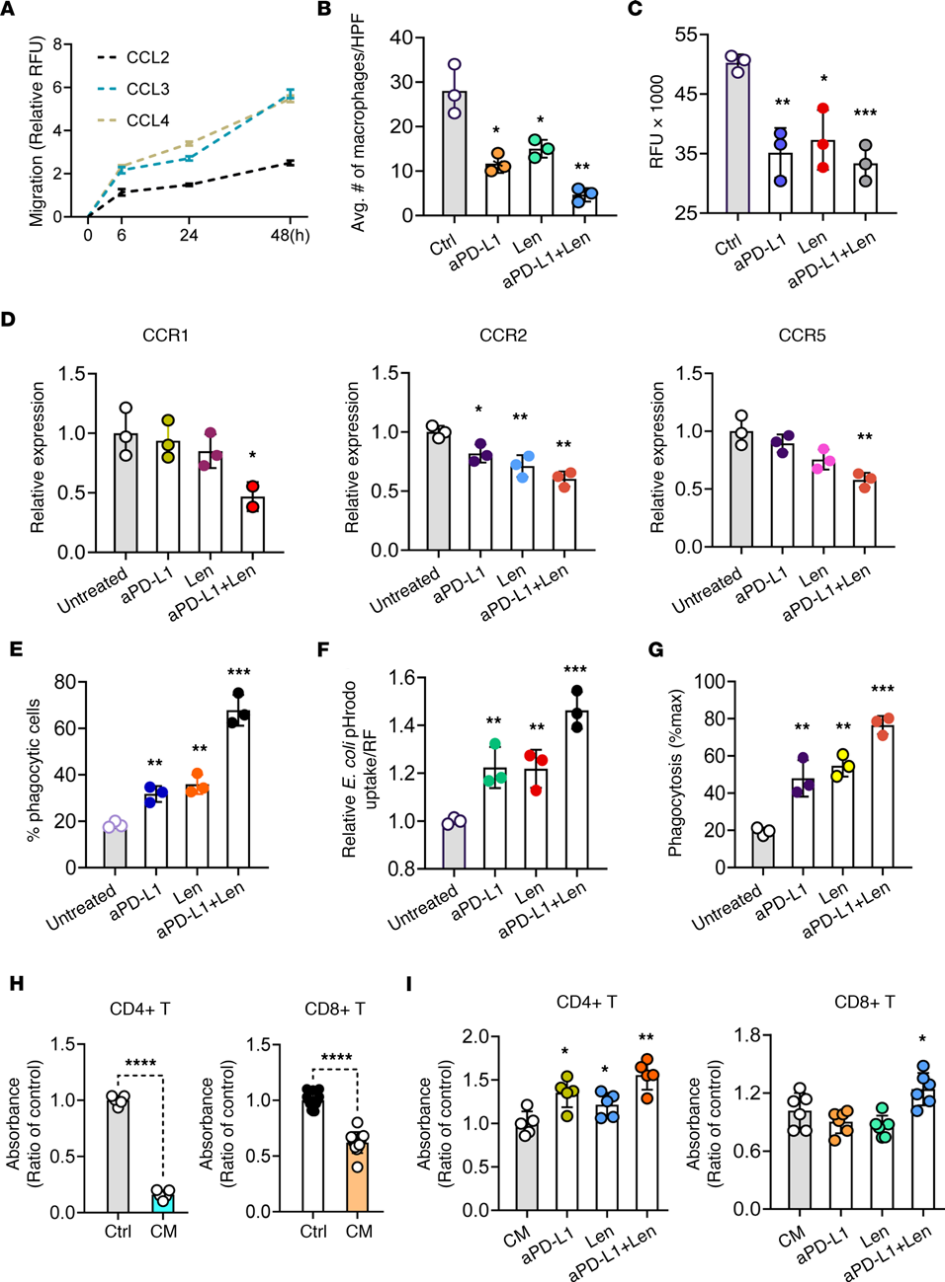
Functional changes of M2-like TAMs after lenalidomide and anti–PD-L1 Ab treatment. (**A**) M2-like TAMs were exposed to CCL2, CCL3, and CCL4 for 6, 24, and 48 hours; migration was determined by QCM 24-well Cell Migration assay (*n* = 3). (**B**) Transwell migration assays were performed on CTCL cell line supernatant-induced TAMs that were untreated or treated with anti–PD-L1, lenalidomide, or their combination (*n* = 3). (**C**) The migration ability of CTCL cell line supernatant-induced TAMs in the same treatment conditions as in **B** were quantified by QCM 24-well Cell Migration assay (*n* = 3). (**D**) The expression levels of chemokine receptors were shown for M2-like TAMs before and after treatment (*n* = 3). (**E**) CTCL cell line supernatant-induced TAMs in the same treatment conditions as in **B** were incubated for 2 hours with fluorescent polystyrene latex beads (*n* = 3). (**F**) CTCL cell line supernatant-induced TAMs in the same treatment conditions as above were incubated with *E*. *coli* for 45 minutes. Uptake of pHrodo Green *E*. *coli* BioParticles Conjugate was analyzed using flow cytometry (*n* = 3). (**G**) A phagocytosis assay with M2-like TAMs showing that anti–PD-L1 and lenalidomide treatment enhanced phagocytosis of cancer cells (*n* = 3). (**H**) CD4^+^ or CD8^+^ T cell proliferations were assessed when they were cocultured with CTCL cell line supernatant-induced TAMs using the MTT assay. ****P < 0.0001 by 2-tailed Student’s *t* test. (**I**) MTT assay was performed for CD4^+^ and CD8^+^ T cell proliferation upon coculture with MyLa-supernatant induced TAMs in the same treatment conditions as above. **B**–**I**, each value is the mean ± SD of 3 independent experiments. **H** and **I** (CD4^+^ T, *n* = 5; CD8^+^ T, *n* = 6). For **B**–**G** and **I**, significant difference was determined by 1-way ANOVA and *P* ≤ 0.05 was considered significant. *P < 0.05; **P < 0.01; ***P < 0.001.

We first evaluated the role of chemokines in the cell migration of M2-like TAMs induced by CTCL cell line supernatant; we performed both the Boyden chamber and CHEMICON QCM assays. The data revealed that CCL2, CCL3, and CCL4 induced M2-like TAM migration in vitro (Figure 6A). Next, we assessed the effects of anti–PD-L1, lenalidomide, and their combination on the cell migration of M2-like TAMs induced by CTCL cell line supernatant. The Boyden chamber assay demonstrated that anti–PD-L1 (*P* < 0.01) and lenalidomide (*P* < 0.05) decreased M2-like cell migration and, when used in combination, showed synergistic effects (*P* < 0.01) (Figure 6B). The results were further validated by the CHEMICON QCM Cell Migration Assay, which also indicated that anti–PD-L1 and lenalidomide synergistically inhibited M2-like TAM migration (*P* < 0.001) (Figure 6C). Notably, treatment with anti–PD-L1 (10 μg/mL), lenalidomide (10 μM), or their combination did not affect M2-like TAM viability as measured by 3-(4,5-dimethylthiazol-2-yl)-2,5-diphenyl-tetrazolium bromide (MTT) assay over a 72-hour period (Supplemental Figure 12). To explore the mechanism of anti–PD-L1, lenalidomide, and their combination on cell migration, we performed flow cytometry to evaluate CCR1, CCR2, and CCR5 expression before and after treatment. We found that the expression of CCR1 (*P* < 0.05), CCR2 (*P* < 0.01), and CCR5 (*P* < 0.01) on M2-like TAMs was significantly inhibited when treated with both anti–PD-L1 and lenalidomide (Figure 6D). Our findings suggest that CCLs interact with their corresponding CCRs on M2-like TAMs to facilitate their migration. Furthermore, we investigated whether anti–PD-L1 and lenalidomide would enhance phagocytosis of M2-like TAMs cultured in MyLa-induced conditioned medium and coincubated with fluorescent latex beads (Figure 6E), pHrodo *E*. *coli* BioParticles (Figure 6F), or CTCL cells (Figure 6G). In M2-like TAMs treated with anti–PD-L1 (*P* < 0.01) and lenalidomide (*P* < 0.01), the levels of phagocytic macrophages increased when compared with untreated control, and combining both drugs demonstrated a synergistic effect (*P* < 0.001) (Figure 6, E and F). Both anti–PD-L1 (*P* < 0.01) and lenalidomide (*P* < 0.1) effectively induced M2-like TAM phagocytosis against CTCL cells compared with untreated control, but the combination performed significantly better than each alone (*P* < 0.001, Figure 6G). We assessed the ability of anti–PD-L1 and lenalidomide to stimulate T cell proliferation using T cells isolated from healthy PBMCs and cocultured with M2-like TAMs. Our results indicated that proliferation of untreated CD4^+^ and CD8^+^ T cells was inhibited when cocultured with M2-like TAMs induced in CTCL cell line supernatant (*P* < 0.0001, Figure 6H). While treatment with anti–PD-L1 alone had no effect, and lenalidomide showed only mild effects, combining both drugs significantly augmented the proliferation of CD4^+^ T cells (*P* < 0.01) and CD8^+^ T cells (*P* < 0.05, Figure 6I).

Taken together, our results indicated that immunosuppressive M2-like TAMs induced by CTCL in vitro show impaired phagocytic ability and inhibited T cell proliferation. Anti–PD-L1 combined with lenalidomide induced functional changes in TAMs, thereby enhancing phagocytic activity and impairing migration of M2-like TAMs and augmenting T cell proliferation to improve antitumor immunity.

## Discussion

Recent studies have demonstrated that the skin microenvironment plays an important role in the development and progression of the CTCL variants MF and SS (7–9); however, the precise nature of relevant immune cell populations involved in CTCL pathogenesis is still unclear. Here, we aimed to investigate the role of TAMs within the CTCL microenvironment. The hematology B37 database highlights that CTCL has the highest CD163^+^ cell type among the listed hematologic malignancies. In our study, we showed that the numbers of CD163^+^ dermal macrophages are increased compared with that in healthy control, which was consistent with previous reports (8, 9, 29). In vivo, Wu et al. found that abrogated tumor formation by depletion of macrophages in a murine cutaneous lymphoma model suggested a central role of macrophages in CTCL pathogenesis (9). Recent studies indicated that CD163 is also a possible marker for predicting immune-related adverse events caused by immune checkpoint inhibitors (30, 31). We found that the CD163^+^ M2-like TAMs in lesional skin of CTCL were positive for PD-1 expression, and the number of PD-1^+^CD163^+^ cells was significantly higher than in healthy controls, suggesting that PD-1^+^CD163^+^ M2 phenotypes have important roles in the development of CTCL. In vitro, we induced PD-1^+^ M2-like TAMs that produced high levels of IL-10, rendering the environment even more immunosuppressive, and we investigated whether M2-like phenotypes could be reprogrammed or reshaped with anti–PD-L1 (durvalumab) and lenalidomide. We found that both PD-1 and PD-L1 were downregulated after anti–PD-L1 treatment and enhanced by lenalidomide, possibly through autocrine or paracrine mechanisms of PD-L1–mediated changes of the cytokine milieu. In addition, the potently decreased expression of IL-10 after lenalidomide or combined lenalidomide /anti–PD-L1 treatment may have contributed to PD-1/PD-L1 downregulation, since IL-10 was shown to strongly induce PD-1 and PD-L1 expression on immune cell subsets (32).

Because TAMs sequentially differentiate from monocytes into functional macrophages, they have heterogeneity and plasticity in cancer. Monocytes recruited from the circulation differentiate into tissue macrophages and are primed by several cytokines, such as IL-4 and IL-13. Thereafter, macrophages change their functional phenotype in response to their environmental stimuli (33, 34). Macrophages have a variety of functions, such as phagocytosis, migration, and cytokine production, and play important roles in various skin diseases. CTCL is an immunosuppressive environment, and we therefore investigated chemokines and cytokines relevant to M2-like TAM migration and polarization. We first showed that CCL3 and CCL4 levels were significantly increased and correlated with the infiltration of CD163^+^ cells in CTCL. In parallel, the overexpression of Th2 cytokines IL-4 and IL-13 in lesional CTCL skin specimens and plasma (35, 36) correlated with TAM accumulation in CTCL. This widespread imbalance in cytokines may reflect the underlying loss of immune function and the Th2 phenotypic origin of the malignant T cells (36). In our study, the heatmap of immune cell clusters showed that CCL2, CCL3, and CCL4 and CCR1, CCR2, CCR3, and CCR5 were all associated with an M2-like macrophage profile, although their selected expression in other immune cells was also seen. The findings suggest that the chemokines are relevant for M2-like TAMs in the CTCL TME, thereby recruiting circulating monocytes and promoting their differentiation into tissue macrophages to promote an immunosuppressive environment in CTCL. Notably, our study demonstrated that the anti–PD-L1 Ab and lenalidomide inhibited migration of M2-like TAMs by affecting interactions between chemokines and chemokine receptors to prevent a TAM-mediated immunosuppressive environment.

Signaling interactions create a permissive environment for the expression of immune checkpoints. Importantly, this study and recent reports indicated that NF-κB and JAK-STAT pathways are involved in CTCL pathogenesis and progression (37–39). Earlier in vivo and in vitro experiments demonstrated that Th2 cytokines induce M2 polarization via the transcription factor STAT6 (40) and that IL-17 induces a polarized M2 phenotype via NF-κB (41). In other malignancies, TAMs are mainly characterized as M2-like TAMs, which show a typical M2 receptor expression and functional profile (42). However, the molecular mechanisms controlling macrophage polarization in CTCL are not well understood. Our study revealed increased levels of the IKK complex of the NF-κB signaling pathway and the activation of the JAK3/STAT pathway. We found high expression of JAK3, STAT1, STAT3, STAT5, and STAT6 in CTCL cell line supernatant-induced TAMs. While we and others have found that STAT3 activation confers high PD-1 and/or PD-L1 expression in CTCL tumor cells and other NK/T cell malignancies that may promote tumor immune evasion (43), our data specifically demonstrated that JAK/STAT and NF-κB *pathways* are major mediators of PD-1 expression in M2-like TAMs in CTCL. Previously published data on PD-1^+^ TAMs in a mouse model of cancer correlated with decreased phagocytic activity against tumor cells (19). In our study, the anti–PD-L1 Ab durvalumab demonstrated an antitumor immune response in CTCL by downregulation of both JAK/STAT and NF-κB pathways, driving PD-1^+^ M2-TAM polarization toward a more proinflammatory M1-like phenotype and inducing phagocytosis in vitro, which was enhanced by lenalidomide. Furthermore, PD-1^+^ M2-like TAM–impaired effector T cell proliferation was reversed, possibly through downregulation of PD-1/PD-L1, allowing the stimulation of T cells.

Collectively, our study indicated that PD-1 was overexpressed on CTCL-infiltrating M2-like TAMs associated with their immunosuppressive role. Our findings critically highlight the effects of anti–PD-L1/lenalidomide on TAMs as a potential strategy to target the CTCL TME that can be explored for other T cell malignancies, including subtypes of CTCL.

## Methods

### Human samples

Sections of 45 FFPE skin biopsies of CTCL lesions (Table 1) and 3 healthy samples (patients undergoing reconstructive plastic surgery served as healthy controls) identified from City of Hope’s tissue repository were used for mRNA expression analysis (15). All patients were staged according to the revised staging and classification of CTCL (44).

Human peripheral blood samples from HDs were obtained from the City of Hope blood donor center. T cells were enriched by EasySep Human CD4^+^ and CD8^+^ T cell Isolation Kits (catalog 19052, catalog 19053). CD14^+^ monocytes were enriched by EasySep Human Monocyte Isolation Kit (catalog 19059) and subsequently cultured in MyLa or Hut78 supernatant for 72 hours.

### Cell culture

MyLa and Hut78 cells were purchased from American Type Culture Collection (ATCC) and cultured in RPMI 1640 (Corning, catalog 10-040-cv) or IMDM (Gibco, catalog 12440053), supplemented with 10% FBS (Corning, catalog 35-010-CV), 100 IU/mL penicillin and 100 μg/mL (Gibco, catalog 15140122) streptomycin in a humidified, 5% CO_2_ incubator at 37°C. The cell lines were tested for mycoplasma contamination using a PCR Mycoplasma detection kit (ABM, catalog G238).

### Luminex cytokine analysis

Representative plasma samples originated from 45 patients with CTCL with skin samples used for RNA-Seq and supernatants of CTCL cell line cultures were analyzed for 30 cytokines using a Cytokine Thirty-Plex bead-linked immunoassay (Invitrogen, catalog LHC6003M) as per the manufacturer’s protocol. Luminex beads were read by a Flexmap 3D Luminex system, and cytokine concentrations were calculated using Bio-Plex Manager 6.2 software with a 5-parameter curve-fitting algorithm applied for standard curve calculations.

### Multiplex immunofluorescence staining and evaluation

FFPE sections of CTCL were subjected to anti–PD-1 (Origene, catalog UM800091; 1:3,000), anti–PD-L1 (Abcam, catalog SP142; 1:600), and anti-CD163 (Invitrogen, catalog 10D6; 1:600) for multiplex immunofluorescence staining as previously described (15). Images were acquired with a Vectra 3 microscope, and different channels were separated with Inform Image/Cell Analysis software (ver 2.3). The expression of CD163^+^PD-1^+^, CD163^+^PD-L1^+^, and PD-1^+^PD-L1^+^ cells in digitized multiplex fluorescent IHC images was quantified using CellProfiler (45). The denominator of the percentage of positive cells are DAPI-positive cells. χ^2^ tests for proportions were performed in R.

### Migration assays

#### Boyden chamber migration assay.

Macrophages were resuspended in a serum-free medium, counted, and diluted to a concentration of 1 × 10^5^ cells/mL. The 24-well Millicell hanging cell culture inserts (MilliporeSigma, catalog MCEP24H48; 8 μm pore) were seeded with 100 μL of macrophage suspensions untreated or pretreated with durvalumab (anti–PD-L1, 10 μg/mL), lenalidomide (10 μM), or a combination of both. The plates were incubated for 4 hours at 37°C in humidified air with 5% CO_2_. After the incubation period, cells were fixed in 4% paraformaldehyde for 15 minutes at room temperature. Nonmigrated cells were removed with a cotton swab. Cells were stained with 0.5% crystal violet solution for 15 minutes. Migratory or invasive cells were counted under an optical microscope (Olympus CX41), and the results of 6 high-power fields were averaged.

#### QCM chemotaxis cell migration assay.

Cell migration was assessed using the QCM Chemotaxis Cell Migration Assay Kit (MilliporeSigma, catalog ECM506) according to the manufacturer’s instructions. In brief, 0.5 × 10^6^ macrophages/mL in serum-free media were placed in the inner wells of migration chambers, and the outer well of the chambers was charged with or without anti–PD-L1, lenalidomide, or drug combination in 10% FBS media. The kits were incubated at 37°C for 4 hours. After media removal, trypsin lysis buffer/trypan blue dye solution was added to each well, and the plates were incubated for 15 minutes at room temperature. The results were quantified using a Cytation 5 Cell Imaging Multi-Mode Reader (BioTek Instruments) at a 480/520 nm filter set.

### Phagocytosis assays

#### Flow cytometry–based phagocytosis assay.

A phagocytosis assay was performed using a Phagocytosis Assay Kit (IgG-FITC) from Cayman Chemical (catalog 500290) according to the manufacturer’s instructions. Briefly, latex beads coated with rabbit IgG-FITC complex (Cayman Chemical, catalog 400291) were added directly to M2-like TAMs that were induced with CTCL cell line supernatant to a final dilution of 1:100 to 1:500 for 2 hours. Cells were washed with assay-supplied buffer, stained with anti-human CD163 (BioLegend, catalog 333608, PerCP-Cy5.5) Ab to gate the total macrophages, and transferred to FACS tubes for flow cytometry (Fortessa, BD Bioscience). Phagocytosis was analyzed using FlowJo 10.6.2 software program (Tree Star)

#### Phagocytosis of pHrodo E. coli.

Macrophages were incubated with pHrodo Green *E*. *coli* BioParticles Conjugate (Thermo Fisher Scientific, catalog P35366). Cells were collected after 45 minutes’ incubation at 37°C and 5% CO_2_ and stained with Abs to M2-like TAM surface markers (CD163, PerCp-Cy5.5). The pretreated macrophages’ uptake of pHrodo Green *E*. *coli* BioParticles Conjugate was analyzed using the Fortessa (BD Biosciences).

#### Phagocytosis of CTCL cells.

CTCL cell line supernatant-induced M2-like TAMs were harvested and equally divided into FACS tubes with 1 × 10^5^ cells per tube. Target CTCL cells were labeled with cell-permeant calcein AM as previously described (46). M2-like TAMs were stained with the anti-CD163 Ab conjugated with APC. The phagocytosis index was quantified as the percentage of macrophages that phagocytosed cancer cells during the incubation (the ratio of GFP^+^APC^+^ cells to APC^+^ cells). For pretreatment experiments, cancer cells or macrophages were pretreated with anti–PD-L1 and/or lenalidomide followed by phagocytosis assays. Phagocytosis index was normalized to the maximal response in the experiments.

### ELISA

Supernatants were tested for the secretion of IL-1β, CXCL-10, CXCL-11, and IL-10 before and after treatment by ELISA. The following ELISA kits were used: human IL-1β/IL-1F2 QuicKit ELISA kit (R&D Systems, catalog QK201), human CXCL10/IP-10 Quantikine QuicKit ELISA kit (R&D Systems, catalog QK266), human CXCL11/I-TAC Quantikine ELISA Kit (R&D Systems, catalog DCX110), and human IL-10 Quantikine ELISA Kit (R&D Systems, catalog D1000B). The SpectraMax iD3 (Molecular Devices) multimode microplate reader was used, and all experiments were performed in triplicates.

### T cell proliferation assay

CD4^+^ or CD8^+^ T cells from HDs were cocultured with CTCL cell line supernatant-induced M2-like TAMs (CD4^+^/CD8^+^ T cells and TAMs were generated from the same donor) for 72 hours with or without anti–PD-L1 and/or lenalidomide treatment. Afterward, T cell proliferation was assessed by MTT assay (Abcam, catalog ab211091), and the absorbance was quantified at 490 nm in a Cytation 5 Cell Imaging Multi-Mode Reader (BioTek Instruments).

### Flow cytometry

CTCL cell line supernatant-induced M2-like TAMs were stained with anti-human CD80 (BioLegend, catalog 305236, BV711), anti-human CD163 (BioLegend, catalog 333608, PerCP-Cy5.5), anti-human CD206 (SONY Biotechnology, catalog 2205600, APC-Cy7), anti-human PD-1 (TONBO Biosciences, catalog 50-210-3431, PE), anti-human PD-L1 (BioLegend, catalog 393610, APC), anti-human CD191 (CCR1) (BioLegend, catalog 362904, PE), anti-human CD192 (CCR2) (BioLegend, catalog 357208, APC), anti-human CD195 (CCR5) (BioLegend, catalog 359120, FITC), and isotype control antibodies and analyzed on a Fortessa flow cytometer. The FlowJo 10.6.2 software program (Tree Star, Inc.) was used for data analysis.

### mRNA quantification by RT-qPCR

Total RNA was prepared using TriZol reagent (Invitrogen), and cDNA synthesis was performed using the High-Capacity cDNA Reverse Transcription Kit (Applied Biosystems, catalog 4368814). Quantitative real time PCR (RT-qPCR) was performed with TaqMan Fast Advanced Master Mix (Thermo Fisher Scientific, catalog 4444964) by TaqMan method with CFX96 Touch Real-Time PCR Detection System (Bio-Rad). The following primers were used: OAZ1 (Hs00427923_m1), IL-1β (Hs01555410_m1), CXCL-10 (Hs00171042_m1), CXCL-11 (Hs00171138_m1), and IL-10 (Hs00961622_m1); all reactions were performed in triplicate.

### Western blot

Following treatment, cells were lysed, and total protein concentration was measured by colorimetry using a BCA kit (Thermo Fisher Scientific, catalog 23225), after which 25 μg of protein was separated by 4%–15% mini-Protean TGX gel (Bio-Rad) and transferred to a nitrocellulose membrane (Bio-Rad) using Trans-Blot Turbo System (Bio-Rad). Proteins underwent IB with the following Abs: JAK3 (Cell Signaling Technology, catalog 8827S), STAT1 (Cell Signaling Technology, catalog 9172S), p-STAT3 (Cell Signaling Technology, catalog 9131S), STAT5 (Cell Signaling Technology, catalog 94205S), STAT6 (Cell Signaling Technology, catalog 5397S), SOCS2 (Cell Signaling Technology, catalog 2779S), SOCS6 (Abcam, catalog ab197335), NF-κB Pathway Sampler Kit (Cell Signaling Technology, catalog 9936), and GAPDH (Cell Signaling Technology, catalog 5174S). Primary Abs were detected by binding goat anti-rabbit IgG–HRP (Cell Signaling Technology, catalog 7074) and visualized by the enhanced chemiluminescence method (Thermo Fisher Scientific, catalog 32209).

### RNA-Seq bioinformatics analysis

RNA-Seq was performed by the City of Hope Integrative Genomics Core facility using Illumina Hiseq 2500 as previously described (47). RNA-Seq libraries were prepared using the KAPA Hyperprep RNA-Seq kit following the manufacturer’s recommendations. The raw sequences were quality filtered and aligned to hg19 using TopHat v2. Reads per kilobase of transcript per million mapped reads were calculated. The counts were normalized, and differential expression analyses were conducted using the Bioconductor package edgeR. Multiple comparisons were adjusted using the FDR ≤ 0.05. Further gene expression correlation analysis and correlation of clinicopathology patient phenotype with genomics data were performed using Partek Flow software, v10.0.22.0504 (48). The Ingenuity Pathway Analysis (IPA) canonical pathway analysis were performed with the use of QIAGEN IPA v73620684 (QIAGEN, https://digitalinsights.qiagen.com/IPA) (49). The public cohort mining used Hematology_B37_20220115_v14 by selecting disease of interest and was performed on OmicSoft Studio software version 10.0.1.81. (QIAGEN) (50). To analyze immune cell proportions in CTCL lesional samples, formatted data were uploaded to the CIBERSORT web portal (https://cibersort.stanford.edu/). The proportions of the immune cell characteristics from each sample were determined by using the CIBERSORT R package. CIBERSORT (51) was used to analyze the relative expression levels of genes in individual tissue samples according to their gene expression profiles to predict the proportion of immune cell characteristics in each tissue.

### Statistics

All analyses were performed using GraphPad Prism 9 software (Graphpad Software). Comparison between groups was analyzed by unpaired 2-tailed Student’s *t* test (between 2 groups) or 1-way ANOVA (among 3 or more groups) unless otherwise indicated. Spearman’s correlation coefficient was used to investigate the relationships between gene expression. Data in the study were shown as the mean ± SD unless otherwise noted. *P* values greater than 0.05 were considered NS; *P* ≤ 0.05 was considered significant.

### Study approval

Ethical approval for the study was obtained from the Institutional Review Board at City of Hope, in accordance with the Declaration of Helsinki. Written informed consent was received prior to participation on the IRB 15185 protocol.

### Data availability

All the data sets were deposited in the NCBI’s Gene Expression Omnibus (GEO) repository under the accession number GSE113113.

## Author contributions

ZH, HQ, and CS performed experiments, analyzed and interpreted data, performed statistical analyses, and/or designed figures. ZH, JFS, and CQ generated the manuscript. JZ and CQ acquired data and provided patient data; XW and HQ generated and analyzed RNA-Seq data and provided figures. YCY, LM, EH, MF, DS, and PPL contributed to methodology, data analysis, and experimental design. ZH, STR, and CQ conceptualized the experimental design, supervised the study, and revised the manuscript with input from all authors.

## Supplementary Material

Supplemental data

## Figures and Tables

**Table 1:**
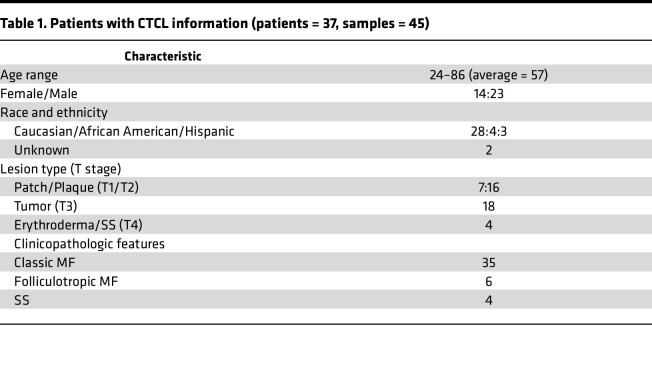
Patients with CTCL information (patients = 37, samples = 45)

## Version 1 07/10/2023

Electronic publication
