# Supplementary material for: Reprogramming of PD-1+ M2-like tumor-associated macrophages with anti–PD-L1 and lenalidomide in cutaneous T cell lymphoma
Source: JCI Insight. 2023 Jul 10;8(13):e163518. doi: 10.1172/jci.insight.163518 (PMC10371344; doi:10.1172/jci.insight.163518)
Supplement: Supplemental data [file jciinsight-8-163518-s091.pdf]

Supplemental materials and methods

MTT assay

The MTT staining method (Abcam, catalog Ab211091) was used for cell viability and proliferation. After 72-hour treatment period by anti-PD-L1, lenalidomide, or their combination, 50 µl of MTT reagent was added to each well and the plate was further incubated at 37°C for 3 hours. Thereafter the medium was aspirated, and the wells washed with PBS, 150 µl of MTT solvtion was added to each well. The plate was wrapped in foil and placed on a shaker for 15 minutes. The absorbance was determined at 590 nm on a SpectraMax ID3 Microplate Reader (Molecular Devices, LLC., San Jose, USA).

Supplementary figures and legends

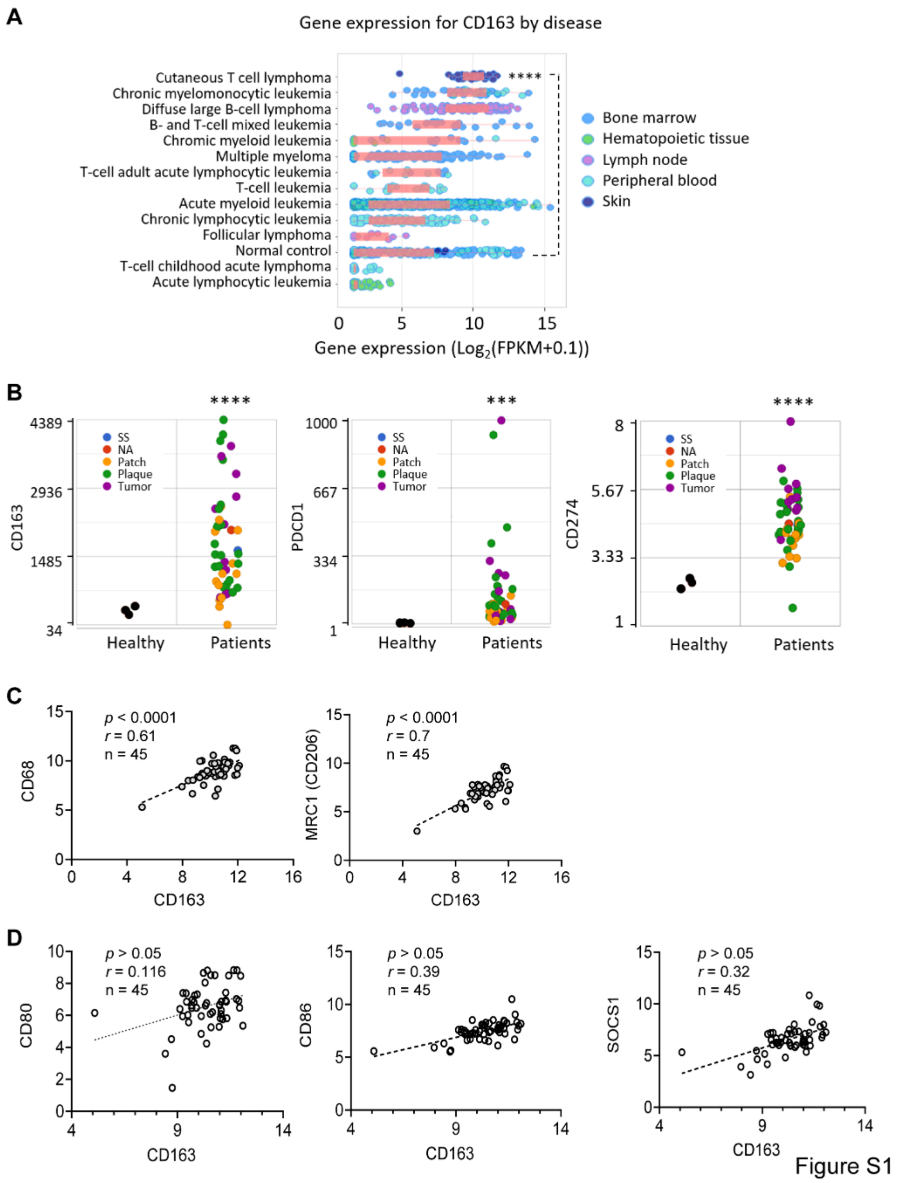

Figure S1. The different immune cell contents in patch, plaque, tumor, and SS stage of CTCL. (**A**) Transcript RPKM (Reads Per Kilobase per Million mapped reads) for the *CD163* gene, organized by tumor type. Statistical differences were calculated by one-way ANOVA (****$p <$ 0.0001). (**B**) RNA-seq gene-level analysis plots showing the expression levels of *CD163*, *PDCD1* and *CD274* genes in CTCL (n = 45) and normal controls (n = 3). Yellow dots indicate patch, green dots show plaque, purple dots indicate tumor, blue dots indicate SS, and black dots represent normal. Data are representative of 3 independent experiments. Two-tailed Student t-test (***p < 0.001, ****p < 0.0001). **C-D**, Pearson correlation between *CD68*, *MRC1* (CD206) (C), and *CD80*, *CD86, SOCS1* (D) gene expression with *CD163* (n =45). Data are representative of 3 independent experiments. The Spearman's correlation coefficient was determined (**C:** r = 0.61, ****p < 0.0001; r = 0.7, ****p < 0.0001; **D:** r = 0.116, p > 0.05; r = 0.39, p > 0.05; r = 0.32, p > 0.05).

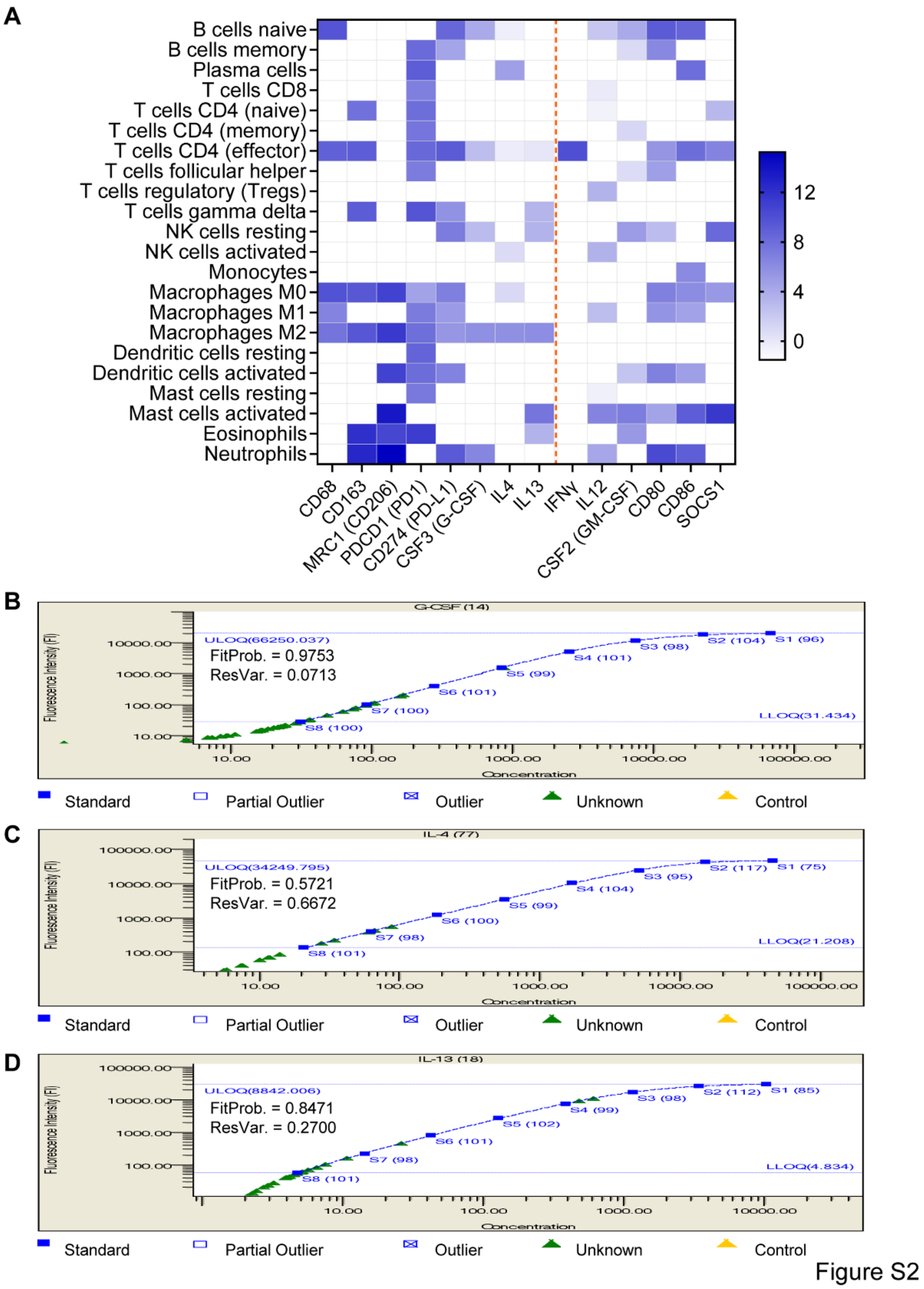

Figure S2. Cellular origin of cytokines and M2-like macrophage marker in CTCL patients. A. CIBERSORT analysis was performed to identify the cellular origin of CSF3, IL4, IL13, IFNγ, IL12, CSF2 and immune cell–expressing CD68, CD163, MRC1 (CD206), PDCD1, CD80, CD86, and SOCS1 (n = 45). Data are representative of 3 independent experiments. **B-D.** Standard curves of cytokines were generated using Luminex cytokine assay.

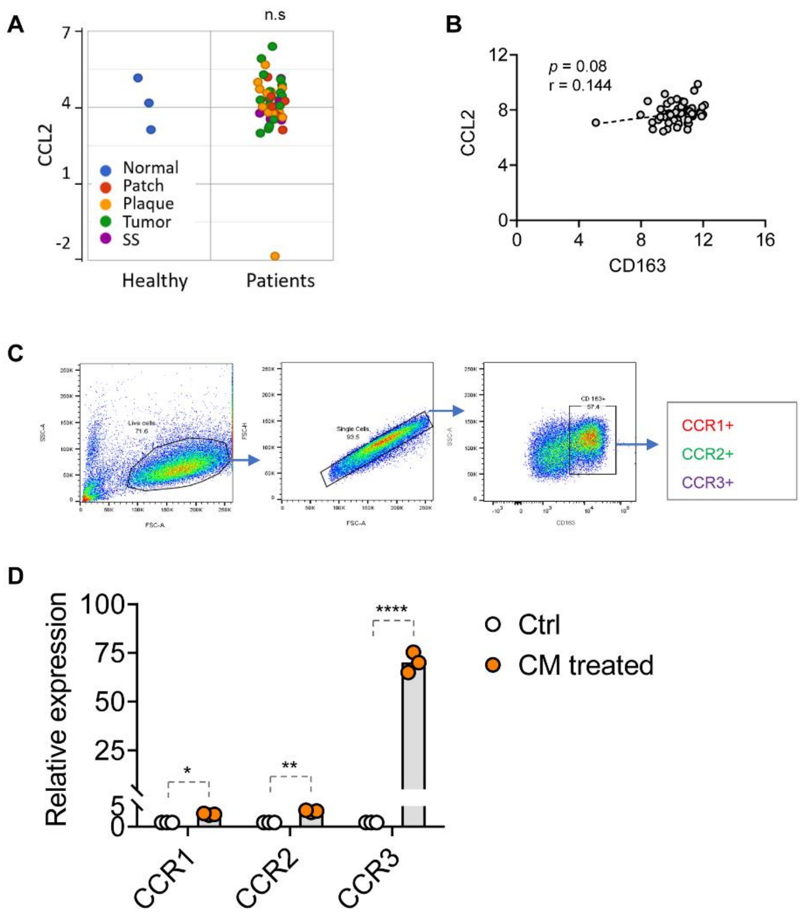

Figure S3

Figure S3. Cellular origin and level of chemokines in CTCL patients. (**A**) RNA-seq gene-level analysis plots indicated the expression levels of the *CCL2* gene in CTCL (n = 45) and normal controls (n = 3). The red dots show patch, yellow dots show plaque, green dots indicate tumor, and purple dots indicate SS, whereas the blue dots represent normal. Data are representative of 3 independent experiments. Statistical differences were calculated by the two-tailed Student t-test, $p \leq 0.05$ indicated statistical significance, n.s: not significant. (**B**) Pearson correlation between *CCL2* gene expression from CTCL patients and *CD163* (n = 45). Data are representative of 3 independent experiments. Spearman's correlation coefficient, $p \leq 0.05$ indicated statistical significance. (**C**) The gating strategy used for chemokine receptor expression on macrophages cultured with and without CTCL cell line supernatant. (**D**) The quantitative expression of chemokine receptors on macrophage cultured with and without CTCL cell line supernatant (n = 3). Data are representative of 3 independent experiments. Two-tailed Student t-test (*$p < 0.05$, **$p < 0.01$, ****$p < 0.0001$).

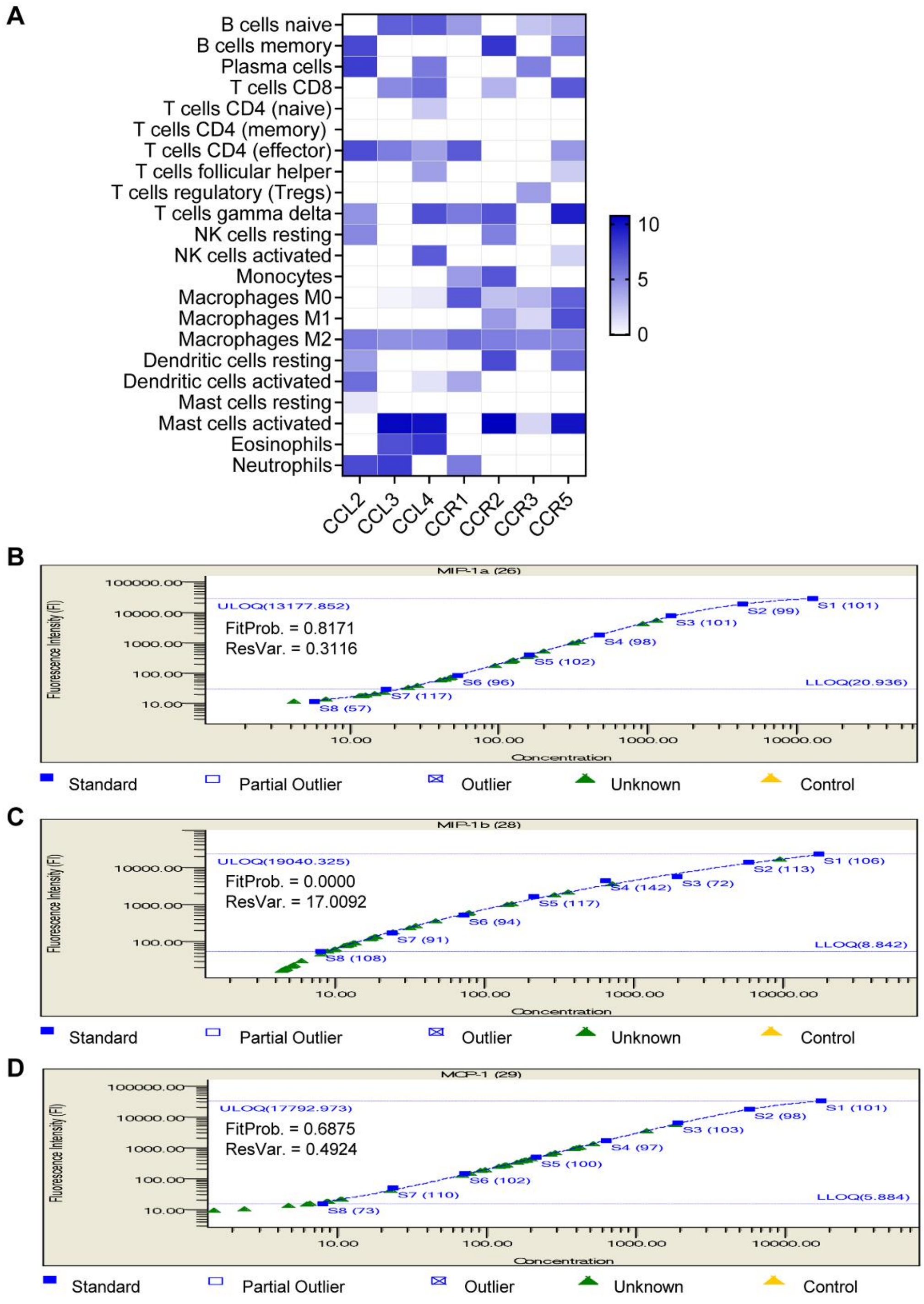

Figure S4

Figure S4. Cellular origin of chemokines on M2-like macrophage marker in CTCL patients. (**A**) CIBERSORT analysis was performed to identify the cellular source of CCL2, CCL3, CCL4 and immune cell–expressing CCR1, CCR2, CCR3, and CCR5 (n = 45). Data are representative of 3 independent experiments. **B-D.** Standard curves of chemokines were generated using Luminex cytokine assay.

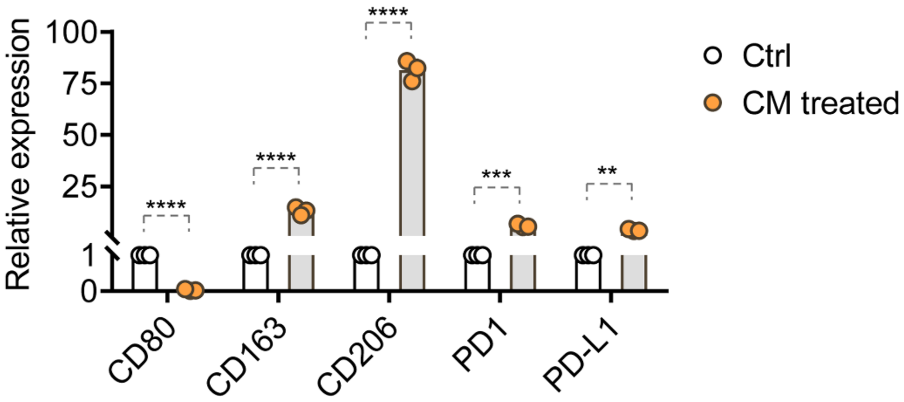

Figure S5

Figure S5. The quantitative expression of CD80, CD163, CD206, PD1, and PD-L1 in TAMs induced with or without CTCL cell line supernatant were detected using flow cytometry. Data are representative of 3 independent experiments with mean ± SD, n = 3. Two-tailed Student t-test (***p* < 0.01, ****p* < 0.001, *****p* < 0.0001).

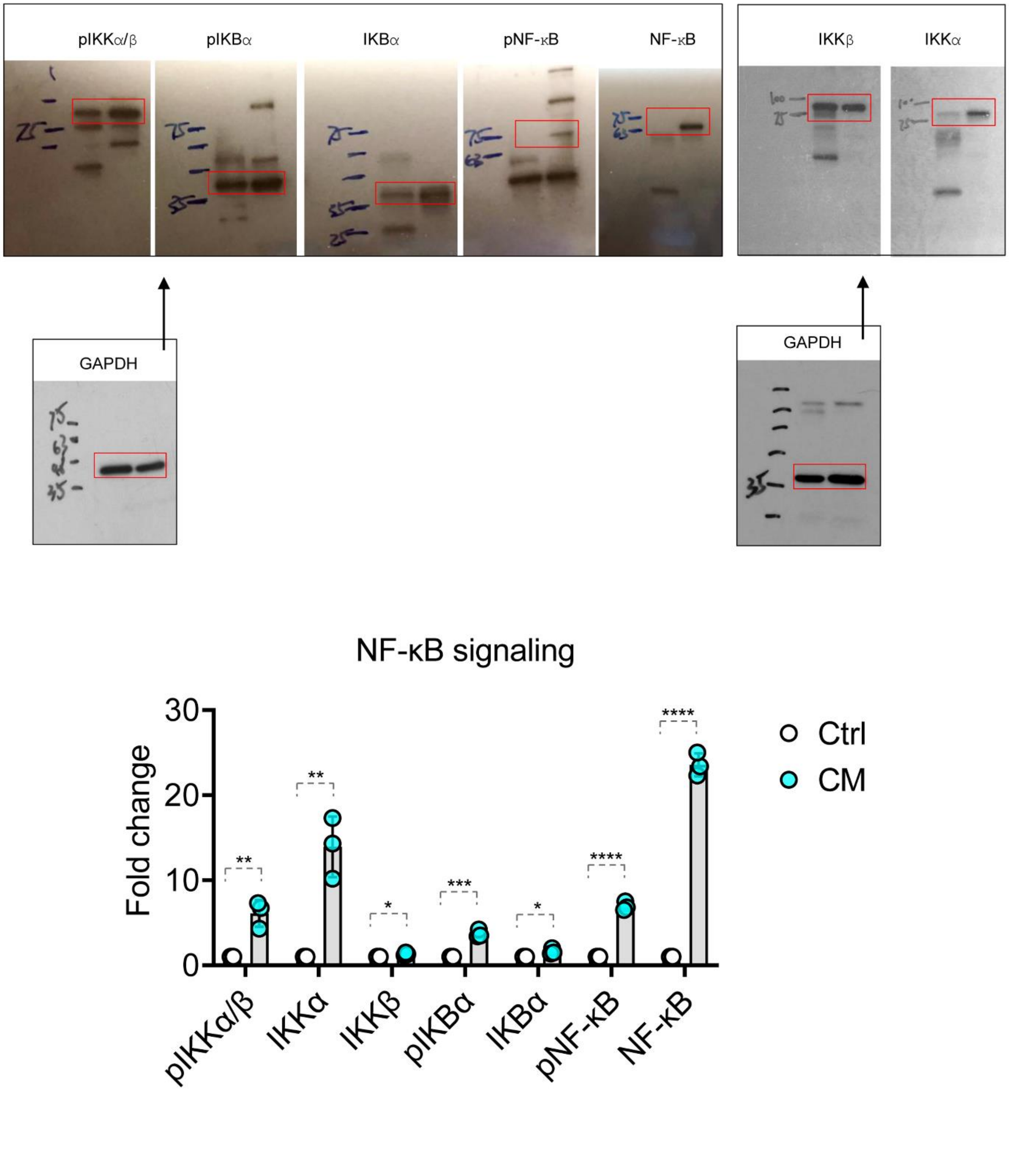

Figure S6

Figure S6. Unedited Western blot bands of the expression of pIKKα/β, IKKα, IKKβ, pNF-κB, IKBα, pIKBα, NF-κB and GAPDH running at different times. The quantitative protein levels of NF-κB signaling were detected by Western blot in TAMs treated with or without CTCL cell line supernatant. Data are representative of 3 independent experiments with mean ± SD, n = 3. Two-tailed Student t-test (*$p < 0.05$, **$p < 0.01$, ***$p < 0.001$, ****$p < 0.0001$).

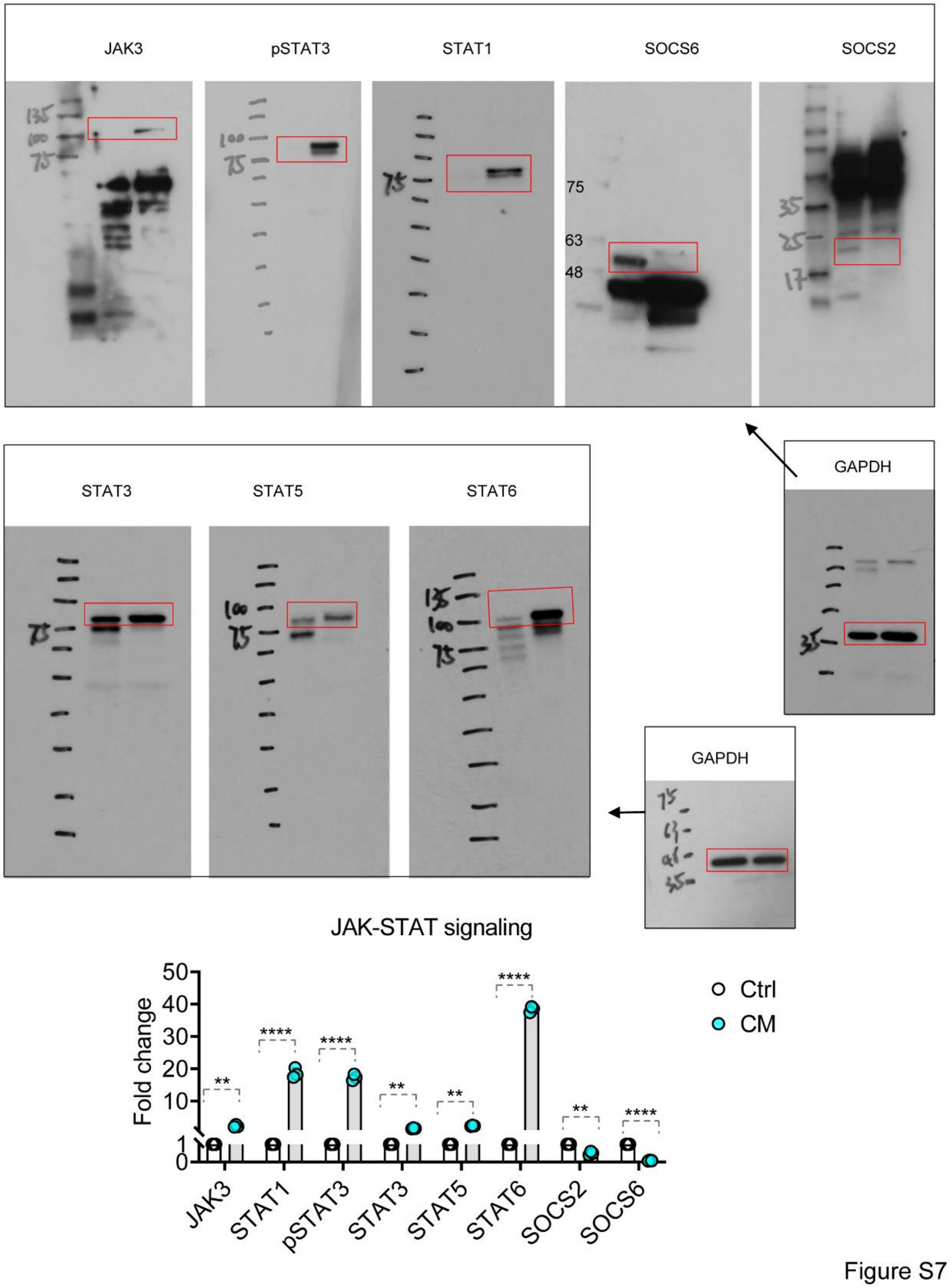

Figure S7. Unedited Western blot bands of the expression of JAK3, STATs, SOCSs and GAPDH running at different times. The quantitative protein levels of JAK-STAT signaling were measured by Western blot in TAMs treated with or without CTCL cell line supernatant. Data are representative of 3 independent experiments mean ± SD, n = 3. Two-tailed Student t-test (***p* < 0.01, *****p* < 0.0001).

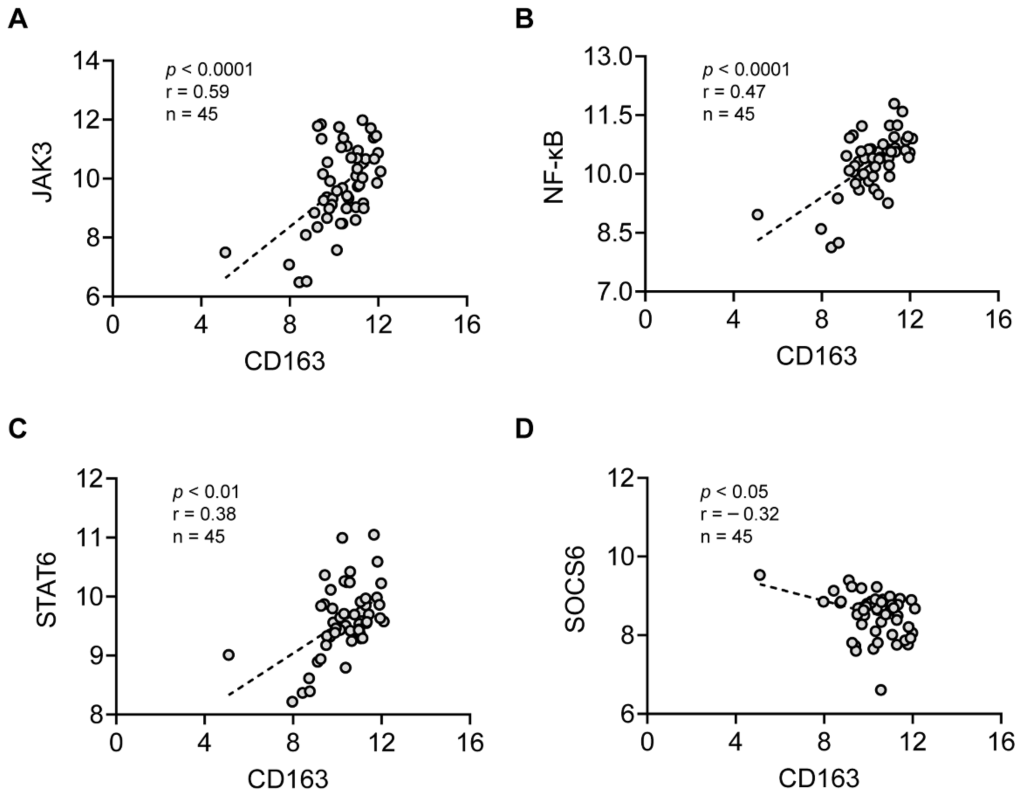

Figure S8

Figure S8. Association between CD163 and the expression of other genes of interest in CTCL lesional skin. A-D, Correlation analysis was used to determine the relationship between *CD163* and *JAK3* (**A**), *NF-κB* (**B**) *STAT6* (**C**), and *SOCS6* (**D**). Data are representative of 3 independent experiments. Spearman's correlation coefficient, $p \leq 0.05$ indicated statistical significance, n = 45.

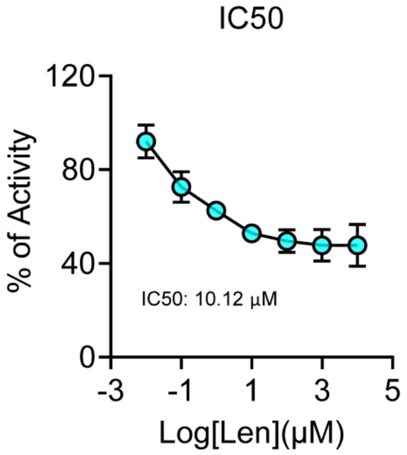

Figure S9

Figure S9. ***IC_{50}*** **of lenalidomide on M2-like macrophages.** Each value is the mean ± SD of three independent experiments. Non-linear regression analysis, n = 3.

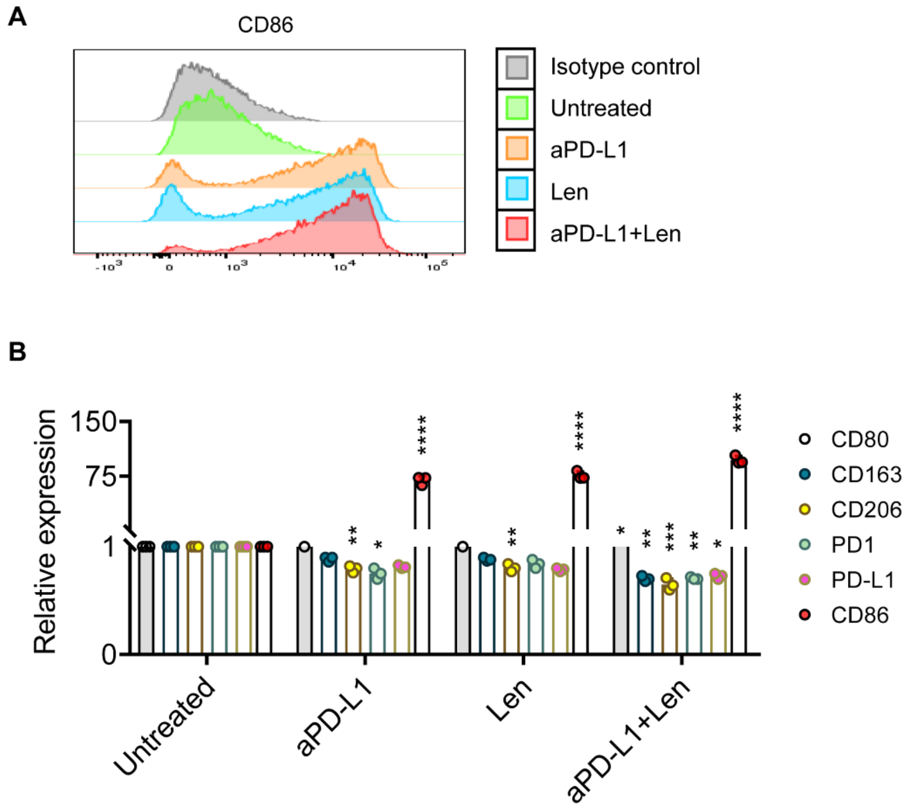

Figure S10

Figure S10. Lenalidomide and anti-PD-L1 antibody reprogram M2-like TAMs *in vitro*. (**A**) CTCL cell line supernatant-induced TAMs were treated with anti-PD-L1 (10 µg/ml), lenalidomide (10 µM), or their combination for 72 hours. The expression of CD86 was detected by flow cytometry. The histograms are representative of three independent experiments. The shadow is the fluorescence intensity of the isotype control. (**B**) The quantitative expression of CD80, CD86, CD163, CD206, PD1, and PD-L1 were detected in TAMs treated with anti-PD-L1 (10 µg/ml), lenalidomide (10 µM), or their combination using flow cytometry. Significant difference was determined by one-way ANOVA and $p \leq 0.05$ indicated statistical significance, n = 3. (*$p < 0.05$, **$p < 0.01$, ***$p < 0.001$, ****$p < 0.0001$).

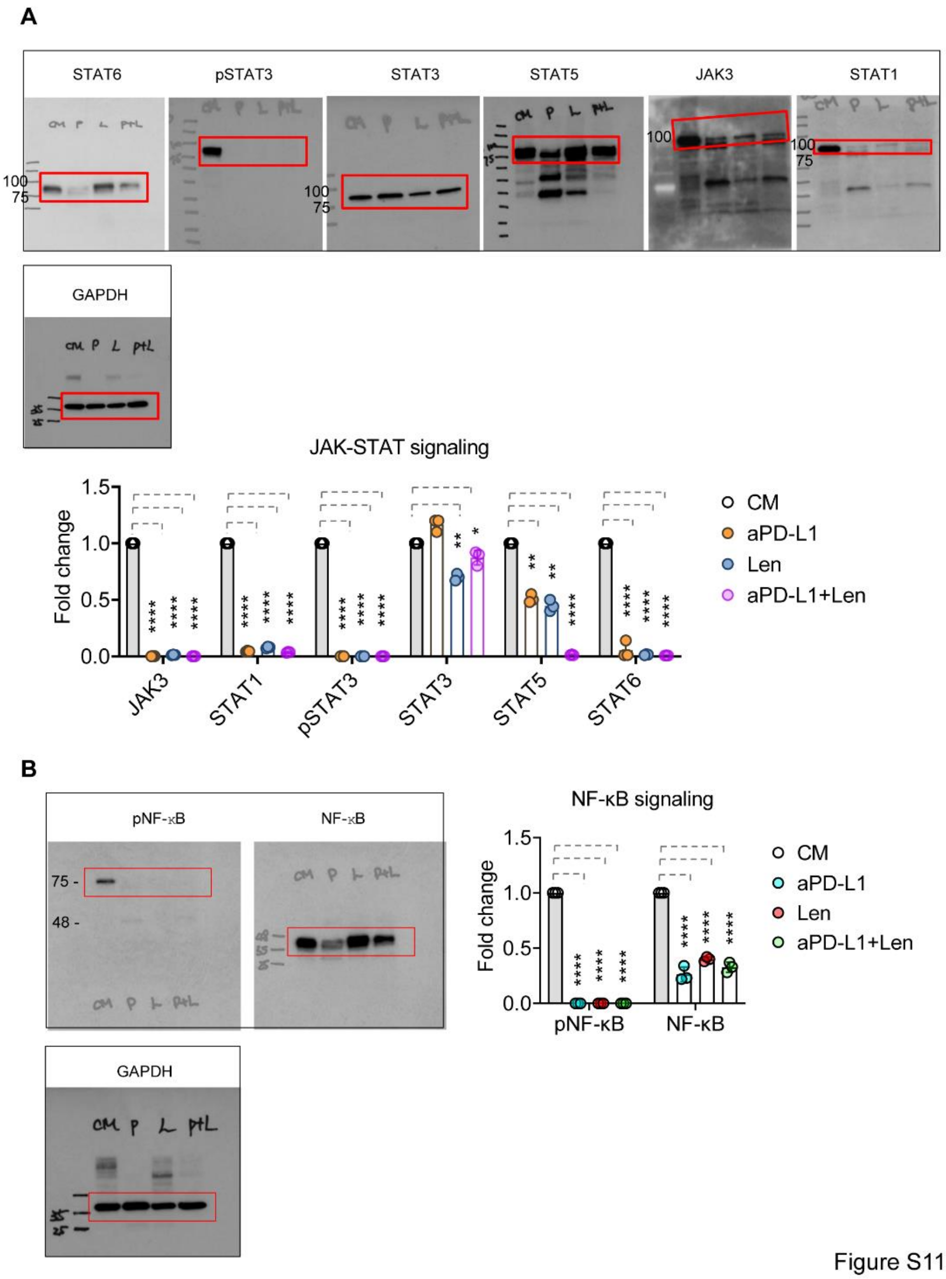

Figure S11

Figure S11. Unedited Western blot bands of the expression of JAK-STAT signaling and GAPDH running at different times. The quantitative protein levels of NF-κB signaling and JAK-STAT signaling were measured by Western blot in TAMs treated with anti-PD-L1, lenalidomide, or their combination. (**A**) The quantitative protein levels of NF-κB signaling were detected by Western blot in TAMs treated with anti-PD-L1, lenalidomide, or their combination. Unedited Western blot bands of the expression of NF-κB signaling and GAPDH running at different times. (**B**) The quantitative protein levels of JAK-STAT signaling were detected by Western blot in TAMs treated with anti-PD-L1, lenalidomide, or their combination. Data are representative of 3 independent experiments with mean ± SD for **A** and **B**. Significant difference was determined by one-way ANOVA and $p \leq 0.05$ indicated statistical significance.

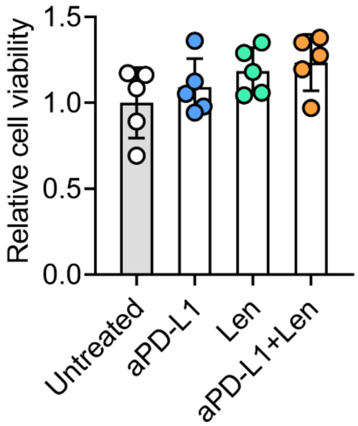

Figure S12

Figure S12. M2-like macrophage viability assessed by MTT assay. M2-like macrophages were treated with anti-PD-L1 (10 µg/ml), lenalidomide (10 µM), or their combination for 72 hours. Cell viability was measured in response to anti-PD-L1, lenalidomide or their combination treatment. Data are presented as mean ± SD from 3 biological replicates, n = 5. Data are representative of 3 independent experiments with mean ± SD. Significant difference was determined by one-way ANOVA and $p \leq 0.05$ indicated statistical significance.
